# New Pathogenesis Mechanisms and Translational Leads Identified by Multidimensional Analysis of Necrotizing Myositis in Primates

**DOI:** 10.1128/mBio.03363-19

**Published:** 2020-02-18

**Authors:** Priyanka Kachroo, Jesus M. Eraso, Randall J. Olsen, Luchang Zhu, Samantha L. Kubiak, Layne Pruitt, Prasanti Yerramilli, Concepcion C. Cantu, Matthew Ojeda Saavedra, Johan Pensar, Jukka Corander, Leslie Jenkins, Lillian Kao, Alejandro Granillo, Adeline R. Porter, Frank R. DeLeo, James M. Musser

**Affiliations:** a Center for Molecular and Translational Human Infectious Diseases Research, Department of Pathology and Genomic Medicine, Houston Methodist Research Institute and Houston Methodist Hospital, Houston, Texas, USA; b Department of Pathology and Laboratory Medicine, Weill Cornell Medical College, New York, New York, USA; c Department of Microbiology and Immunology, Weill Cornell Medical College, New York, New York, USA; d Department of Mathematics and Statistics, Helsinki Institute of Information Technology, University of Helsinki, Helsinki, Finland; e Department of Biostatistics, University of Oslo, Oslo, Norway; f Comparative Medicine Program, Houston Methodist Research Institute, Houston, Texas, USA; g Department of Surgery, University of Texas McGovern Medical School, Houston, Texas, USA; h Department of Internal Medicine, Houston Methodist Research Institute and Houston Methodist Hospital, Houston, Texas, USA; i Laboratory of Bacteriology, Rocky Mountain Laboratories, National Institute of Allergy and Infectious Diseases, National Institutes of Health, Hamilton, Montana, USA; GSK Vaccines

**Keywords:** bacterial pathogenesis, bacterial virulence, dual RNA-seq, necrotizing fasciitis, pathogen-host interaction, *Streptococcus pyogenes*

## Abstract

A fundamental goal of contemporary biomedical research is to understand the molecular basis of disease pathogenesis and exploit this information to develop targeted and more-effective therapies. Necrotizing myositis caused by the bacterial pathogen Streptococcus pyogenes is a devastating human infection with a high mortality rate and few successful therapeutic options. We used dual transcriptome sequencing (RNA-seq) to analyze the transcriptomes of S. pyogenes and host skeletal muscle recovered contemporaneously from infected nonhuman primates. The *in vivo* bacterial transcriptome was strikingly remodeled compared to organisms grown *in vitro*, with significant upregulation of genes contributing to virulence and altered regulation of metabolic genes. The transcriptome of muscle tissue from infected nonhuman primates (NHPs) differed significantly from that of mock-infected animals, due in part to substantial changes in genes contributing to inflammation and host defense processes. We discovered significant positive correlations between group A streptococcus (GAS) virulence factor transcripts and genes involved in the host immune response and inflammation. We also discovered significant correlations between the magnitude of bacterial virulence gene expression *in vivo* and pathogen fitness, as assessed by previously conducted genome-wide transposon-directed insertion site sequencing (TraDIS). By integrating the bacterial RNA-seq data with the fitness data generated by TraDIS, we discovered five new pathogen genes, namely, S. pyogenes 0281 (*Spy0281* [*dahA*]), *ihk-irr*, *slr*, *isp*, and *ciaH*, that contribute to necrotizing myositis and confirmed these findings using isogenic deletion-mutant strains. Taken together, our study results provide rich new information about the molecular events occurring in severe invasive infection of primate skeletal muscle that has extensive translational research implications.

## Abstract (precis)

Necrotizing myositis caused by Streptococcus pyogenes has high morbidity and mortality rates and relatively few successful therapeutic options. In addition, there is no licensed human S. pyogenes vaccine. To gain enhanced understanding of the molecular basis of this infection, we employed a multidimensional analysis strategy that included dual RNA-seq and other data derived from experimental infection of nonhuman primates. The data were used to target five streptococcal genes for pathogenesis research, resulting in the unambiguous demonstration that these genes contribute to pathogen-host molecular interactions in necrotizing infections. We exploited fitness data derived from a recently conducted genome-wide transposon mutagenesis study to discover significant correlation between the magnitude of bacterial virulence gene expression *in vivo* and pathogen fitness. Collectively, our findings have significant implications for translational research, potentially including vaccine efforts.

## INTRODUCTION

Molecular interaction between a pathogen and its host can result in asymptomatic carriage and disease and, sometimes, death. Regardless of the ultimate outcome, an important aspect of pathogen and host interaction is that changes in gene transcription shape complex phenotypes such as site of infection, pathogen dissemination, and disease severity. Thus, a fuller understanding of the infection cycle and disease mechanisms requires analysis of molecular events occurring contemporaneously in a pathogen and host.

Streptococcus pyogenes, also known as group A streptococcus (GAS), is a strict human pathogen responsible for >700 million infections and ∼517,000 deaths annually worldwide (1). This organism causes diverse infections that range in severity from relatively mild conditions such as pharyngitis to life-threatening septicemia and necrotizing fasciitis/myositis (2). GAS has been classified historically based on sequence variation in the *emm* gene encoding M protein, a cell-surface molecule that is antiphagocytic and an important virulence factor (3). Whereas the majority of invasive infections in North America and Europe are caused by a relatively small number of *emm* types, for example, *emm1*, *emm3*, *emm12*, and *emm28* (4–11), strains of these serotypes are relatively rare in high-disease-burden, low-income regions. Despite a century of effort by many investigators, there is no licensed vaccine available to protect humans against GAS disease.

GAS is a primary cause of bacterial necrotizing fasciitis/myositis, an infection that is associated with remarkably high rates of morbidity and mortality. Many different M protein types of GAS can cause deep-tissue necrotizing infections, but strains of M1 are especially prominent. As with many GAS infections, the molecular pathogenesis of necrotizing myositis is complex, with many bacterial molecules implicated. Research over many years has shown that a broad array of secreted and surface-displayed virulence molecules contribute to infection pathogenesis, including but not limited to M protein, cytolytic toxins such as streptolysin O and streptolysin S, degradative enzymes (e.g., streptococcal pyrogenic toxin B cysteine protease, C5a peptidase, and an IgG-cleaving enzyme), and a hyaluronic acid capsule. Although progress has been made in understanding mechanisms contributing to necrotizing infections, much remains to be learned.

To further vaccine and other translational research efforts, several studies have been conducted to elucidate virulence determinants expressed *in vivo* during different types of infections (12–16). Initial studies measuring gene expression during the interaction of GAS with its host were performed with hybridization approaches that involved custom-made Affymetrix gene chips (17–20). For example, Graham et al. (17) studied GAS genes expressed during experimental soft tissue infection in mice. In addition, in a large study involving 20 nonhuman primates (NHPs), Virtaneva et al. (20) described GAS gene transcript changes that occurred over a 84-day infection cycle that involved initial colonization of the upper respiratory tract (URT), clinical pharyngitis, and subsequent asymptomatic carriage. Similarly, Shea et al. (18) analyzed the complicated relationship between changes in GAS and host NHP transcripts over the URT infection cycle. These and other studies have provided much new data about GAS interactions with the host during invasive and URT infection, but knowledge gaps remain.

Dual transcriptome sequencing (RNA-seq) (21) has afforded the ability to determine transcriptome changes using deep-sequencing technologies, and this technique has been exploited using wild-type strains of multiple GAS serotypes and mutant strains in which different virulence regulators have been inactivated (22–28). Recent development of dual RNA-seq analysis has assisted studies designed to measure transcripts of both pathogen and host during infection (29–32). Most dual RNA-seq studies have used host cell lines to analyze pathogen-host interactions (33–40), resulting in new information about the interplay between the two. However, these experiments share the inherent limitation that cell culture infection models exclude the influence of many host factors and thus are limited compared to the intact-animal milieu. A few studies have used mouse infection models of human pathogens, including work done with Pseudomonas aeruginosa (41), Staphylococcus aureus (42), Clostridium perfringens (43), and Yersinia pseudotuberculosis (44). However, dual RNA-seq work has not been conducted with an NHP experimental infection model and a human-restricted pathogen.

In the present study, we used a well-described NHP model of necrotizing myositis (27, 45, 46) and dual RNA-seq to study the relationship between pathogen and host transcripts. Until now, a limited number of studies have been conducted using dual RNA-seq under *in vivo* conditions (41, 42, 47–53). In a very recent study, dual RNA-seq was performed on human tissue biopsiy samples obtained from necrotizing soft tissue infections (54). Here, the GAS RNA-seq data were integrated with fitness data generated previously by a genome-wide transposon mutagenesis screen in necrotizing myositis (46) to select genes for further study. We subsequently created isogenic mutant strains and confirmed the pathogenesis role of five genes not previously known to contribute to necrotizing myositis. In the aggregate, use of this multidimensional investigative strategy resulted in discovery of new genes with important roles in virulence during necrotizing myositis in primates.

## RESULTS

### Altered transcript regulation of GAS during deep skeletal muscle infection in NHPs.

We first examined the relationship between the transcript profiles of GAS strains grown in rich Todd-Hewitt broth supplemented with yeast extract (THY) (classified as grown *in vitro*) and organisms isolated from infected skeletal muscle tissue from NHPs (classified as grown *in vivo*). We hypothesized that the transcript pattern of GAS recovered from infected NHP skeletal muscle tissue is significantly different from the transcript pattern of bacteria grown *in vitro*. This matter is important because the data could be exploited to identify previously undefined genes and mechanisms used by GAS during deep-muscle necrotizing invasive disease.

**FIG 1:**
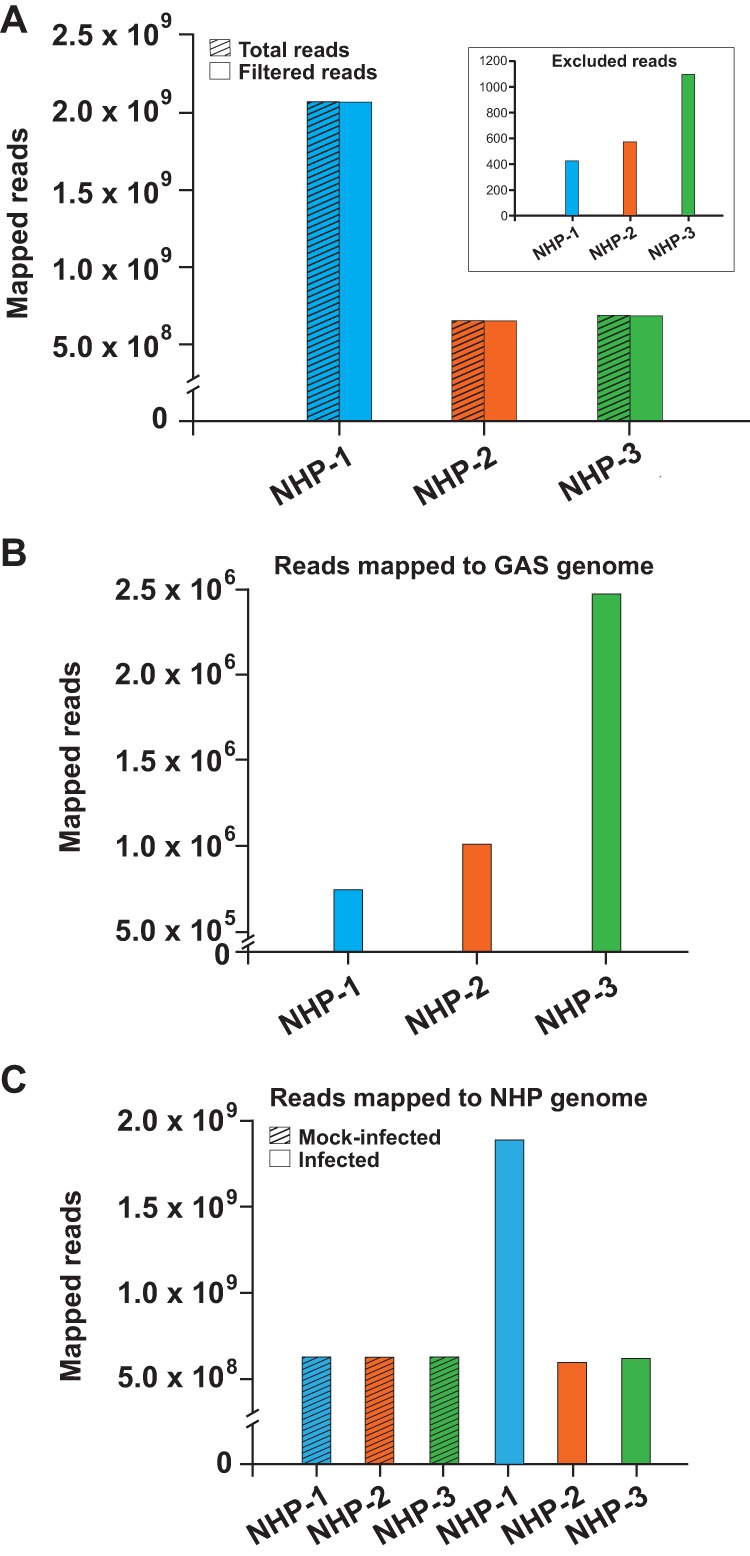
Mapping of transcript reads from *in vivo* samples to GAS and NHP genomes. Numbers of transcript reads per sample are shown. (A) Total number of sequence reads corresponding to each of the three NHPs (hatched columns). Reads simultaneously mapping to both NHP and GAS genomes were excluded (inset). Solid columns represent filtered reads, calculated by subtracting excluded reads from the total number of transcript reads. (B) Number of transcript reads mapping to the reference GAS genome (strain MGAS2221) corresponding to each of the three NHPs. (C) Number of transcript reads corresponding to each of the three NHPs mapping to the NHP genome (http://useast.ensembl.org/Macaca_fascicularis/Info/Annotation). The number of reads mapping to either the GAS genome (panel B) or the genome of mock-infected or infected NHPs (panel C), respectively, was 7.0 × 10^5^ (GAS) or 5.8 × 10^8^/1.9 × 10^9^ for NHP-1, 1.0 × 10^6^ (GAS) or 6.2 × 10^8^/5.8 × 10^8^ for NHP-2, and 2.5 × 10^6^ (GAS) or 6.3 × 10^8^/6.1 × 10^8^ for NHP-3.

To address this issue, we infected NHPs with serotype M1 strain MGAS2221, an organism genetically representative of strains causing a recent global pandemic of invasive infections (27). This strain had been used previously in NHP necrotizing myositis studies (46, 55). Sections from infected skeletal muscle tissue and uninfected controls were processed 24 h postinoculation following a standard procedure required by our approved NHP infection protocol (46, 55), and GAS transcript data were obtained by RNA-seq. Between 5 × 10^8^ and 2.1 × 10^9^ total transcript reads were obtained from each animal (Fig. 1A). Reads mapping to both GAS and NHP genomes constituted a negligible proportion and were excluded from further analysis. The distribution of reads mapping either to the GAS genome or to the genome of mock-infected or infected NHPs is shown in Fig. 1B and C. Importantly, there was very high correlation (*R*^2^ range, 0.87 to 0.99) between the biological replicates, comparing either the *in vitro* GAS samples analyzed in triplicate or the pooled *in vivo* GAS samples for each NHP (see Table S1A in the supplemental material). Similarly, the correlation was very high (*R*^2^ range, 0.94 to 0.96) for the infected and mock-infected NHP samples (Table S1A).

10.1128/mBio.03363-19.8 TABLE S1 Differentially expressed GAS genes comparing *in vivo* and *in vitro* samples. (A) Correlation coefficients for transcript data for *in vivo* and *in vitro* replicates. (B) Differentially expressed genes comparing *in vivo* versus *in vitro* ME and ES samples combined. (C) Differentially expressed genes comparing *in vivo* versus *in vitro* ME samples. (D) Differentially expressed genes comparing *in vivo* versus *in vitro* ES samples. (E) Differentially expressed genes encoding transcriptional regulators. (F) Virulence, metal homeostasis, and stress genes differentially expressed *in vivo* compared to both ME and ES phases *in vitro*. (G) Differentially expressed GAS genes comparing pooled sections 1 and 2 to pooled sections 3, 4, and 5. (H) Differentially expressed virulence genes comparing MGAS2221 and MGAS2221 Δ*Spy0281*. (I) Upregulated genes comparing MGAS2221 to MGAS2221 Δ*dahA* and to MGAS2221 Δ*covR*. (J) Locus tag equivalence between MGAS2221 and MGAS5005. Download Table S1, XLSX file, 0.2 MB. Copyright © 2020 Kachroo et al. 2020 Kachroo et al. This content is distributed under the terms of the Creative Commons Attribution 4.0 International license.

Consistent with our hypothesis that the transcriptome of GAS grown *in vivo* in skeletal muscle is substantially different from that of GAS grown *in vitro*, three distinct clusters were identified by principal component analysis (PCA), corresponding to GAS samples from strains grown *in vitro* collected at either the mid-exponential (ME) or the early stationary (ES) growth phase and strains obtained from the infected NHPs (*in vivo*) (see Fig. S1A in the supplemental material). We collected tissue sections in a concentric fashion with respect to the central inoculation site for the purpose of analyzing transcript changes based on spatial distribution (Fig. S2A). The central inoculation site had higher levels of edema, necrosis, and neutrophils and a higher bacterial load than the more distal sections with fewer GAS bacteria, as shown by CFU counts and consistent with previous data (46). When the *in vivo* samples taken from each section were not pooled prior to PCA, tighter clustering was evident only for section 1, corresponding to the central inoculation site (Fig. S1B and C). The NHP samples clustered into two separate groups, corresponding to the infected and mock-infected samples (Fig. S1D). Furthermore, analyzing pooled sections from the three NHPs, the infected and mock-infected sections clustered separately and, as seen in the PCA, there was a higher level of transcriptional variation among the infected sections than among the mock-infected sections (Fig. S1E).

10.1128/mBio.03363-19.2 FIG S1 Transcriptome analysis of GAS grown *in vitro* and *in vivo* and NHP samples. Principal-component analyses of transcriptome data were performed. The fold change value cutoff and adjusted *P* value cutoff were 1.5 and ≤0.05, respectively. (A) *In vitro*-grown GAS strains in triplicate at the mid-exponential (ME) and early stationary (ES) phases of growth and *in vivo*-grown GAS strains collected from three nonhuman primates (NHP). Five samples (sections 1 through 5) collected from each NHP were pooled for analysis. The *in vitro* ME replicates clustered together and grouped distinctly apart from the *in vitro* ES samples, and both the ME and ES replicates grouped apart from the *in vivo* samples. These three distinct clusters are highlighted within ovals. (B) *In vivo*-grown GAS samples are depicted as separate sections. Five sections corresponding to each NHP are highlighted. The sections are color coded, whereas NHPs are indicated by geometric shapes as follows: NHP-1, circles; NHP-2, triangles; NHP-3, squares. (C) *In vivo* grown samples from panel B shown at a larger scale. Phages and mobile genetic elements were excluded from the analysis. (D) Infected and mock-infected samples from three NHPs (NHP-1, blue; NHP-2, red; NHP-3, green). Data from mock-infected samples (hatched squares) and infected animals (nonhatched circles) clustered distantly from each other. (E) Infected (red) and mock-infected (blue) samples separated by sections. Each section was pooled from three NHPs. All sections corresponding to the mock-infected tissue clustered distinctly together, away from sections corresponding to infected tissue. Download FIG S1, EPS file, 0.5 MB. Copyright © 2020 Kachroo et al. 2020 Kachroo et al. This content is distributed under the terms of the Creative Commons Attribution 4.0 International license.

10.1128/mBio.03363-19.3 FIG S2 Sample collection for dual RNA-seq analysis and GAS genes differentially expressed during infection, grouped by functional categories. (A) The samples were processed in concentric sections. Section 1, at the center, corresponds to the inoculation site, and section 5 is the most distal section. The section-to-section borders are idealized and are less discrete than portrayed. Sections from infected tissue are color coded. Section 1 from the uninfected arm muscle corresponds to the inoculation site, where only PBS was injected. (B) Genes differentially expressed (DE) *in vivo* versus *in vitro* in the ME and ES growth phases combined. (C) Genes DE *in vivo* versus *in vitro* in the ME growth phase (nonhatched) and *in vivo* versus *in vitro* in the ES growth phase (hatched). The plots represent the percentage of up- and downregulated genes in each category. Functional categories were obtained from PATRIC (https://www.patricbrc.org/) for serotype M1 reference strain MGAS5005. Upregulated genes are represented in red and downregulated genes in blue. The fold change value cutoff and adjusted *P* value cutoff were 1.5 and ≤0.05, respectively. Download FIG S2, EPS file, 0.5 MB. Copyright © 2020 Kachroo et al. 2020 Kachroo et al. This content is distributed under the terms of the Creative Commons Attribution 4.0 International license.

Inasmuch as the transcriptome groups were separated by PCA, we next used linear discriminant analysis to identify genes that would enable effective differentiation among the groups. We found that essentially every differentially expressed gene was suitable for discriminating among the groups (data not shown). This result is consistent with the clear separation of the groups identified by PCA.

### Distinct GAS transcriptome landscape in NHP necrotizing myositis.

**FIG 2:**
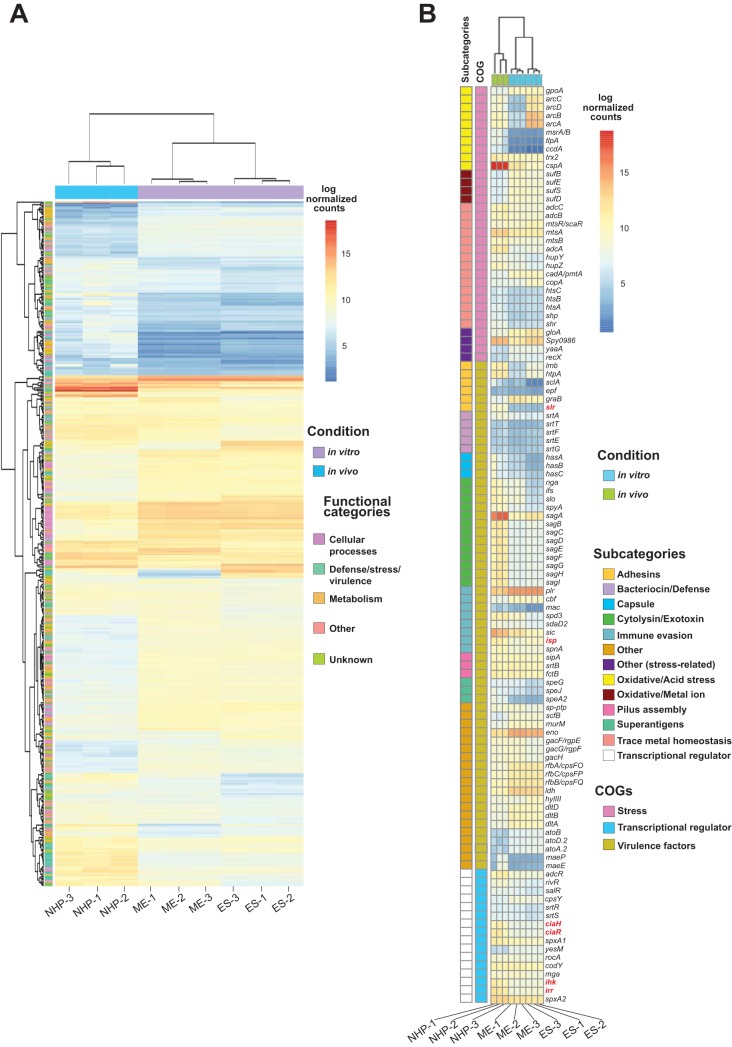
Transcriptome signatures corresponding to GAS strains grown *in vitro* and *in vivo*. The heat map presents log-transformed normalized transcript counts obtained under the following two experimental conditions: (i) GAS grown in rich medium (*in vitro*) and harvested at two phases of growth (ME, mid-exponential; ES, early stationary) and (ii) GAS harvested from infected nonhuman primate (NHP) tissue (*in vivo*). The fold change value cutoff and adjusted *P* value cutoff were 1.5 and ≤0.05, respectively. (A) Heat map showing expression patterns of genes (*n *= 472) found to be significantly differentially expressed between *in vitro* and *in vivo* samples. Color coding is based on log-transformed normalized count values; blue indicates lower transcript expression, and red indicates higher transcript expression. Also indicated is hierarchical clustering of genes (dendrogram along the *y* axis) and of *in vitro* and *in vivo* GAS samples (dendrogram along the *x* axis). (B) Heat map highlighting expression profile of genes (*n *= 110) involved in GAS pathogenesis. Genes are assigned to three broad categories: genes involved in virulence factors, genes encoding transcriptional regulators, and genes involved in responses to stress. Gene names shown in red designate genes subsequently studied in more detail by constructing isogenic deletion-mutant strains.

**FIG 3:**
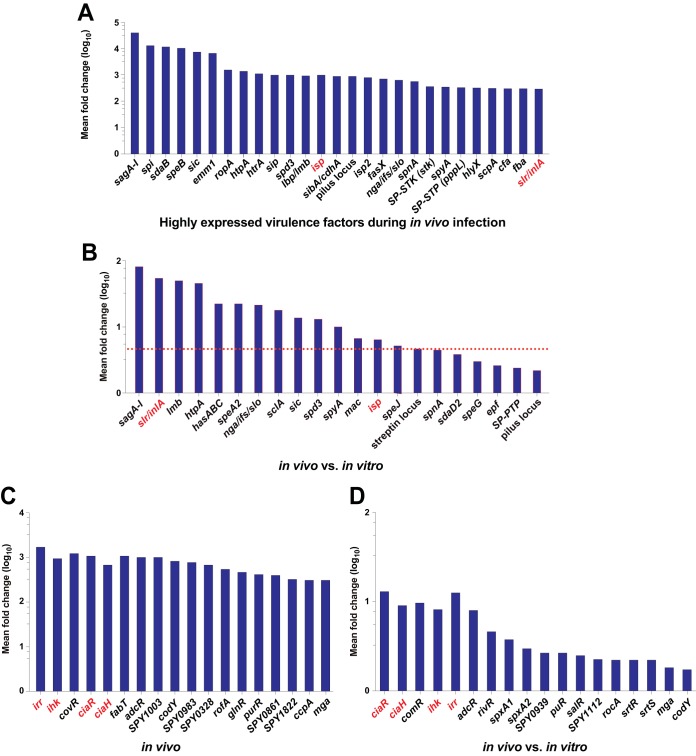
GAS virulence genes highly transcribed during *in vivo* infection and upregulated *in vivo* compared to *in vitro*. (A) Virulence factor genes that are highly transcribed during *in vivo* infection are shown. Log-transformed mean expression (normalized) counts are plotted. Genes are considered highly transcribed if their normalized expression values are in the top 75th percentile. (B) The *y*-axis values represent the mean fold change values determined from combined data from the ME and ES *in vitro* growth phases. Both genes and operons are represented. The horizontal red line corresponds to 5-fold change, an arbitrarily chosen level designed to highlight the magnitude of change. Virulence genes upregulated ≥5-fold are considered to be substantially upregulated. (C) Transcriptional regulators highly transcribed during *in vivo* infection. Log-transformed mean expression (normalized) counts are plotted. (D) Transcriptional factors upregulated during *in vivo* infection compared to *in vitro* growth. Fold change data from *in vivo* versus *in vitro* ME and ES comparisons were averaged, and log-transformed mean log-fold changes are shown (*y* axis). Gene names shown in red designate genes subsequently studied in more detail by constructing isogenic deletion-mutant strains. The fold change value cutoff and adjusted *P* value cutoff were 1.5 and ≤0.05, respectively.

The PCA data indicated that the transcriptome of GAS recovered from the infected NHP skeletal muscle differed markedly from that of GAS grown *in vitro*, regardless of whether the comparator was ME-phase or ES-phase *in vitro*-grown organisms (Fig. 2A) (Table 1; see also Table S1B, C, and D). Although transcript profile differences were identified between each of the two growth phases and *in vivo* GAS, to conserve space, we have concentrated on the description of common themes identified in the two data sets considered in the aggregate (that is, ME and ES *in vitro* versus *in vivo*). Overall, 472 genes (29.8% of 1,584 coding genes, after excluding rRNA, tRNA, and phage genes) were differentially expressed during *in vivo* infection (Fig. 2A), and 92.8% of those 472 genes had concordant differential expression, that is, were regulated in the same direction in the ME and ES phases. Relative to GAS grown *in vitro*, we observed upregulation of genes that encode stress genes, transcriptional regulators, virulence factors, and membrane transporters, among others (Fig. 2B) (Fig. S2B; see also Table S1B, C, and D). In addition, there was substantial alteration of transcripts from genes encoding proteins that would reshape GAS energy metabolism. Highly expressed genes encoding virulence factors and transcriptional regulators during *in vivo* infection are shown in Fig. 3A and C. Genes encoding 52 transcriptional regulators were significantly differentially expressed during *in vivo* compared to *in vitro* infection (Fig. 3D; see also Table S1E). Several known transcriptional regulators that include two-component regulatory systems were highly upregulated, including *adcR* (8-fold), *ciaH* and *ciaR* (11-fold), and *ihk-irr* (11-fold). In addition, the transcript abundances of genes encoding many virulence factors were increased (Fig. 3B; see also Table S1F). For example, 14 genes and/or operons involved in capsule synthesis, cytotoxicity, surface-associated proteins/adhesins, and immune evasion were highly (≥5-fold) upregulated *in vivo*, including the *sagA-I* operon (streptolysin S), *slr* (*Listeria* internalin A homolog), *lmb* (encoding laminin binding protein), *htpA* (encoding a histidine triad protein), *hasABC* (capsule synthesis genes), *speA2*, *nga*/*ifs*/*slo*, *sclA*, *sic*, *spd3*, *spyA*, *mac*, *isp*, *speJ*, the streptin locus, and *spnA* (Fig. 3B). Similarly, genes that are involved in metal ion homeostasis and which have a documented role in pathogenesis, including regulation of iron (*siaABC*, *shr*, *shp*, and *hupYZ*) (56, 57), zinc (*adcRBCA*) (58, 59), and manganese (*mtsAB*) (60), were upregulated (see Table S1F). Genes encoding enzymes in the glycolytic pathway were downregulated, whereas genes encoding enzymes required for the use of alternative carbon sources (Fig. S3A) and for mixed-acid fermentation (Fig. S3B) were upregulated. Finally, genes likely involved in extracellular oxidative stress defense were significantly upregulated (Fig. S3C).

**TABLE 1:** Virulence genes upregulated *in vivo*

MGAS5005 gene designation^*a*^	Gene	Function	MGAS2221 gene designation^*b*^	Fold ME^*c*^	Fold ES^*d*^
Capsule					
*Spy1851*	*hasA*	Hyaluronan synthase	*Spy1825*	11.3	31.3
*Spy1852*	*hasB*	UDP-glucose 6-dehydrogenase	*Spy1826*	12.0	29.2
*Spy1853*	*hasC*	UTP–glucose-1-phosphate uridylyltransferase	*Spy1827*	5.9	10.5

Cytolysin/exotoxin					
*Spy0139*	*nga*	NAD glycohydrolase	*Spy0183*	5.2	35.1
*Spy0140*	*ifs*	Immunity factor for SPN	*Spy0184*	6.1	30.1
*Spy0141*	*slo*	Streptolysin O	*Spy0185*	5.7	27.8
*Spy0351*	*spyA*	c3 family ADP-ribosyltransferase	*Spy0403*	7.4	11.5
*Spy0562*	*sagA*	Streptolysin S precursor	*Spy0598*	121.0	35.1
*Spy0563*	*sagB*	Streptolysin S biosynthesis protein	*Spy0599*	30.8	14.1
*Spy0564*	*sagC*	Streptolysin S biosynthesis protein	*Spy0600*	25.6	13.8
*Spy0565*	*sagD*	Streptolysin S biosynthesis protein	*Spy0601*	27.3	16.9
*Spy0566*	*sagE*	Streptolysin S putative self-immunity protein	*Spy0602*	26.2	17.0
*Spy0567*	*sagF*	Streptolysin S biosynthesis protein	*Spy0603*	21.2	13.7
*Spy0568*	*sagG*	Streptolysin S export ATP-binding protein	*Spy0604*	25.7	16.1
*Spy0569*	*sagH*	Streptolysin S export transmembrane protein	*Spy0605*	32.5	19.1
*Spy0570*	*sagI*	Streptolysin S export transmembrane protein	*Spy0606*	27.2	16.2

Secreted/immune evasion					
*Spy0668*	*mac*	IgG-degrading protease	*Spy0700*	4.6	8.4
*Spy0981*	*cfa*	CAMP factor	*Spy0993*	5.2	NS^*f*^
*Spy1169*	*spd3*	Streptodornase	*Spy1179*	5.3	19.4
*Spy1415*	*sdaD2*	Phage-encoded streptodornase	*Spy1430*	2.9	4.6
*Spy1684*	*ska*	Streptokinase	*Spy0254*	NS	6.3
*Spy1715*	*scpA*	C5A peptidase precursor	*Spy1697*	NS	3.8
*Spy1718*	*sic*	Streptococcal inhibitor of complement	*Spy1699*	7.6	18.5
*Spy1723*	***isp***^*e*^	Immunogenic secreted protein	*Spy1703*	8.4	3.5
*Spy1734*	*spi*	Streptopain protease inhibitor	*Spy1713*	1,080.7	NS
*Spy1735*	*speB*	Streptococcal pyrogenic exotoxin B	*Spy1714*	912.3	NS
*Spy1738*	*spd*	Phage-associated deoxyribonuclease	Spy1717	70.5	NS

Superantigens					
*Spy0182*	*speG*	Exotoxin type G precursor	*Spy0222*	2.1	3.8
*Spy1702*	*smeZ*	Mitogenic exotoxin Z	*Spy0238*	NS	2.6
*Spy0356*	*speJ*	Exotoxin type J precursor	*Spy0408*	4.1	5.9
*Spy0996*	*speA2*	Phage-encoded exotoxin	*Spy1009*	20.4	21.8

Surface-associated/adhesins					
*Spy1711*	*lmb*	Laminin binding protein	*Spy0229*	84.1	8.6
*Spy1710*	*htp*	Streptococcal histidine triad protein	*Spy0230*	80.5	8.5
*Spy1687*	*sclA*	Collagen-like surface protein A	*Spy0251*	NS	26.2
*Spy0561*	*epf*	Putative extracellular matrix binding protein	*Spy0597*	2.4	2.6
*Spy0740*	*fbp*	Fibronectin-binding protein	*Spy0771*	NS	2.1
*Spy1109*	***slr***	Internalin protein	*Spy1122*	54.1	48.6
*Spy1719*	*emm1*	M protein	*Spy1700*	NS	2.1

Transcriptional regulators					
*Spy0947*	***ciaH***	TCS^*g*^ histidine kinase	*Spy0962*	10.2	8.4
*Spy0948*	***ciaR***	TCS response regulator	*Spy0963*	14.0	12.7
*Spy1318*	*rocA*	TCS accessory protein	*Spy1324*	2.3	2.3
*Spy1720*	*mga*	Standalone regulator	*Spy1701*	1.5	2.3
*Spy1724*	***ihk***	TCS histidine kinase	*Spy1704*	10.5	6.5
*Spy1725*	***irr***	TCS response regulator	*Spy1705*	16.1	10.2

Other					
*Spy0281*	***dahA***	Putative role in defense against stress	*Spy0340*	NS	5.0

a Gene designation for MGAS5005.

b Gene designation for MGAS2221.

c Fold differential expression comparing *in vivo* to *in vitro* ME samples.

d Fold differential expression comparing *in vivo* to *in vitro* ES samples.

e Genes highlighted in bold were chosen for isogenic deletion-mutant strain generation.

f NS, not significant.

g TCS, two-component system.

10.1128/mBio.03363-19.4 FIG S3 Differentially expressed GAS genes implicated in carbon metabolism and extracellular oxidative stress defense in GAS. “Spy” numbers correspond to the annotation for serotype M1 reference strain MGAS5005. The fold change value cutoff and adjusted *P* value cutoff were 1.5 and ≤0.05, respectively. (A) Genes encoding glycolytic enzymes are downregulated, and genes involved in transport and utilization of ascorbate, malate, maltose, and glycerol are upregulated. Genes differentially expressed *in vivo*, compared to *in vitro*, at ME and/or ES growth phases are color coded. Upregulated genes are shown in red and downregulated genes in blue. Fold change values are represented in parentheses, with the first value corresponding to *in vivo* versus *in vitro* ME and the second value to *in vivo* versus *in vitro* ES. –, no differential expression. (B) Shift from homolactic fermentation to mixed-acid fermentation inferred from downregulation of *ldh*, encoding lactate dehydrogenase, and upregulation of *lctO*, encoding lactate oxidase, formate transport and assimilation, and mixed-acid fermentation. A reduction in lactate production is likely to result in decreased autoacidification. (C) (Top panel) Schematic of the *ccdA*, *tlpA*, and *msrA* operon in serotype M1 GAS. The transcriptional start site (i) (red arrow) was described previously by Rosinski-Chupin et al. (https://doi.org/10.1186/s12864-019-5613-5). The inferred enzymatic functions of the encoded proteins are indicated. The fold upregulation values *in vivo*, compared to both *in vitro* growth conditions, are depicted in parentheses (ME/ES). –, no differential expression. (Bottom panel) Proposed mechanism for GAS extracellular oxidative stress defense during invasive infections (ii) based on data for Streptococcus pneumoniae. Reducing equivalents (e-) from NADPH are channeled through the thioredoxin system in the cytoplasm and transferred to the outer side of the cell across CcdA. In turn, CcdA transfers the reducing equivalents to the thiol-disulfide oxidoreductase TlpA and through to the bifunctional methionine sulfoxide reductase MsrA. The result is increased protection from oxidation of cysteine residues in secreted proteins and reduction of methionine sulfoxides (iii to v), both likely occurring as a consequence of PMN oxidative burst. This system might also contribute to GAS extracellular protein folding (vi). Download FIG S3, PDF file, 1.0 MB. Copyright © 2020 Kachroo et al. 2020 Kachroo et al. This content is distributed under the terms of the Creative Commons Attribution 4.0 International license.

### Analysis of changes in the NHP transcriptome during GAS invasive infection.

We found that the transcriptomes from the skeletal muscle of NHPs infected with GAS differed substantially from the transcriptomes from mock-infected NHP skeletal muscle (Fig. S1D and E). Compared to mock-infected animals, 1,218 host genes were significantly upregulated and 305 genes were downregulated (Table S2A). Genes encoding proteins involved in the immune response, inflammation, and host defense were significantly upregulated in tissue from infected NHPs (Table S2B). For example, genes encoding cytokines (interleukin-1A [IL-1A], IL-1B, IL-6, IL-7, IL-10, IL-24, and IL-33) and chemokines (CCL11, CCL20, CCL7, CXCL1, CXCL2, CXCL5, CXCL6, CXCL8, and CXCL16) and matrix metalloproteases (MMP1, MMP8, MMP9, and MMP25) were upregulated. We also observed upregulation of genes associated with polymorphonuclear leukocytes (PMNs) or neutrophils, cells that are important components of the early innate immune response. For example, genes encoding proteins involved in recognition of bacterial pathogens, such as pattern recognition receptors (e.g., CD14 and Toll-like receptors [TLRs]); in recruitment of PMNs to the infection site (the gene encoding IL-8 was upregulated by 1,000-fold); and in PMN degranulation and induction of the bactericidal cascade (myeloperoxidase) and inflammatory cascade (IL-1α, IL-1β, IL-1RN, IL-6, IL-7, IL-10, IL-24, IL-33, CCL11, CCL20, CCL7, CXCL1, CXCL2, CXCL5, CXCL6, CXCL8/IL-8, CXCL16, and tumor necrosis factor alpha [TNF-α]), were upregulated, perhaps as a consequence of upregulation of *TREM1* signaling (upregulated 287-fold, *P* = 1.03E–15) and additional pathogen receptors (Table S2A; see also Fig. S4A).

10.1128/mBio.03363-19.5 FIG S4 Pathway analysis of differentially expressed genes in infected NHP tissue and heat map depicting relationship between gene coexpression modules. (A) Top 50 pathways associated with differentially expressed genes in the infected NHP tissue compared to mock-infected tissue. *y* axis data depict –log (*P* value). Pathways with *P* values of ≤0.05 were considered significant. Host pathways containing genes that were upregulated (top panel) or downregulated (bottom panel) during infection are indicated. (B) Heat map depicting similarity of gene coexpression modules (based on eigengene adjacency) among and between the 15-pathogen modules (PM) and 10-host modules (HM) identified by WGCNA (7). Eigengenes are module representatives, and eigengene adjacency is computed based on their correlation. The heat map is colored based on adjacency score as follows: red represents high adjacency (positive correlation), and blue represents low adjacency (negative correlation). GAS gene modules 5 and 6 (marked with an asterisk [*]) were positively correlated with host gene modules 7, 8, and 9 (highlighted in red) and negatively correlated with host gene modules 1 to 4 (highlighted in blue). Download FIG S4, PDF file, 0.4 MB. Copyright © 2020 Kachroo et al. 2020 Kachroo et al. This content is distributed under the terms of the Creative Commons Attribution 4.0 International license.

10.1128/mBio.03363-19.9 TABLE S2 DE genes and significantly enriched biological processes associated with upregulated and downregulated genes comparing infected versus mock-infected NHPs. (A) Differentially expressed genes. (B) Significantly enriched biological processes. Download Table S2, XLSX file, 0.2 MB. Copyright © 2020 Kachroo et al. 2020 Kachroo et al. This content is distributed under the terms of the Creative Commons Attribution 4.0 International license.

A prominent finding observed in the downregulated category was the identification of genes encoding skeletal muscle and connective tissue proteins (Table S2B) such as actin, myosin, tropomyosin, dystrophin, and troponin (Fig. S4A; see also Table S2A). Together, these data are consistent with the substantial degree of tissue destruction and necrotic muscle observed by detailed gross pathological examination of the sections in GAS-infected animals (data not shown).

In addition to protein-coding genes, RNA transcripts for various long intergenic noncoding RNAs (lincRNAs) and microRNAs (miRNAs) were differentially regulated in the infected NHP tissue compared to mock-infected samples (Table S2A). Seven lincRNAs and 12 microRNAs were significantly upregulated (9-fold to 500-fold and 4-fold to 60-fold, respectively), and five (1 lincRNA and 4 miRNA) were downregulated. MicroRNAs such as miR-221, miR-222, and miR-223 were significantly upregulated in the infected host. Although their role in serious invasive GAS infection remains unexplored, they have been reported to be involved in host immune response and inflammation (61–63). Interestingly, microRNAs downregulated in infected host tissue, including miR-30A, miR-133A1, and miR-1 and miR-2, participate in skeletal muscle development and function (64, 65).

We compared the normalized read counts obtained from infected NHPs to the normalized counts of human patient samples from monomicrobial infections caused by S. pyogenes (*n* = 12) recently published by Thänert et al. (54). We found that 87% of the top 100 of the most highly transcribed genes from the infected NHPs were also highly transcribed in the human patient samples. Moreover, among the highly transcribed NHP genes (top 100), 50%, including S100A8, S100A9, CXCL8, HLA-A, NFKBIA, UBC, FPR2, PTX3, and THBS1, were involved in stress response and inflammation. However, a comparison of differential expression results from our study (NHP infected versus mock infected) to their monomicrobial (S. pyogenes) patient data could not be performed due to the unavailability of control samples (i.e., uninfected/healthy tissue) samples in the study by Thänert et al. (54).

To test for a GAS-specific immune response in the host, we analyzed serum biomarkers in infected and uninfected hosts. Of 17 serum biomarkers (cytokines, chemokines, and other immune-related host molecules) tested by immunoassays (see Text S1 in the supplemental material), we found 9 whose levels were significantly increased in GAS-infected NHPs at 24 h postinoculation compared to preinfection values (Fig. S5A). Importantly, the levels of 11 of these biomarkers were significantly increased in serum from humans infected with GAS compared to healthy, uninfected controls (Fig. S5B). Seven serum biomarkers were significantly increased in both NHP and human cohorts. Taken together, these results are consistent with the NHP host transcriptome data and suggest that the NHP model closely mimics the human immune response to invasive GAS infection.

10.1128/mBio.03363-19.1 TEXT S1 List of supplemental tables, study design, additional methods, and supplemental materials references. Download Text S1, DOCX file, 0.1 MB. Copyright © 2020 Kachroo et al. 2020 Kachroo et al. This content is distributed under the terms of the Creative Commons Attribution 4.0 International license.

10.1128/mBio.03363-19.6 FIG S5 Biomarker analysis of serum samples from humans and NHPs. The biomarkers selected for the custom panel were as follows: (i) BAFF/BLyS/TNFSF13B; (ii) CTSS/cathepsin S; (iii) CCL7/monocyte chemoattractant protein-3 (MCP-3)/MARC; (iv) CD30/TNFRSF8; (v) CD44; (vi) CD163; (vii) CXCL2/GRO beta/MIP-2/CINC-3; (viii) Fas/TNFRSF6/CD95; (ix) interferon (IFN) gamma; (x) IL-1 RII; (xi) IL-2; (xii) IL-8/CXCL8; (xiii) MMP-1; (xiv) S100A9; (xv) TNF-α; (xvi) TNF RII/TNFRSF1B; and (xii) TRAIL R3/TNFRSF10C. Red dots/bars, infected NHPs/patients. Blue dots/bars, uninfected NHP/control human serum. (A) Serum samples collected from 17 NHPs prior to inoculation with GAS and at the time of necropsy. The Wilcoxon matched-pair signed rank test was used to compute the *P* values. (B) Serum samples collected from three infected human patients and from nine healthy, uninfected donors. Statistical significance was computed using the Mann-Whitney U test. The *y*-axis data in both panels are represented on a log_10_ scale. Download FIG S5, EPS file, 0.5 MB. Copyright © 2020 Kachroo et al. 2020 Kachroo et al. This content is distributed under the terms of the Creative Commons Attribution 4.0 International license.

### Pathogen-host gene correlation networks.

To investigate possible pathogen-host interactions based on our dual RNA-seq data, we used weighted gene coexpression network analysis (WGCNA) (66) to cluster positively correlated genes into pathogen- and host-specific network modules. On the basis of the normalized transcript counts of the pathogen and the host genes, WGCNA grouped highly correlated genes into clusters/modules. The GAS genes clustered into 15 pathogen modules and the NHP genes into 10 host modules (Table S3A; see also Fig. S4B). A COG enrichment analysis showed that GAS modules 5 and 6 were significantly enriched in genes involved in carbohydrate transport/metabolism (COG category G; *P* value = 0.000) and virulence (COG category V; *P* value = 0.006) and in posttranslation modification (COG category O; *P* value = 0.006) and virulence (COG category V; *P* value = 0.000), respectively (Table S3B). Moreover, an examination of the eigengene network showed that GAS modules 5 and 6 were strongly positively correlated with three host gene modules (modules 7, 8, and 9) involved in immune response, inflammation, and response to stress (Table S3A), a result suggesting that upregulation of GAS virulence genes triggers an immune response in the host. Conversely, GAS modules 5 and 6 were negatively correlated with NHP modules involved in muscle development, contraction, maintenance, and integrity (modules 1 to 4; see Table S3).

10.1128/mBio.03363-19.10 TABLE S3 (A) Functional categories associated with the host gene modules (HM) identified by WGCNA. (B) COG enrichment analysis for pathogen modules (PM). Download Table S3, PDF file, 0.3 MB. Copyright © 2020 Kachroo et al. 2020 Kachroo et al. This content is distributed under the terms of the Creative Commons Attribution 4.0 International license.

### Dual RNA-seq identifies significant differences in spatially distinct transcriptomes.

**FIG 4:**
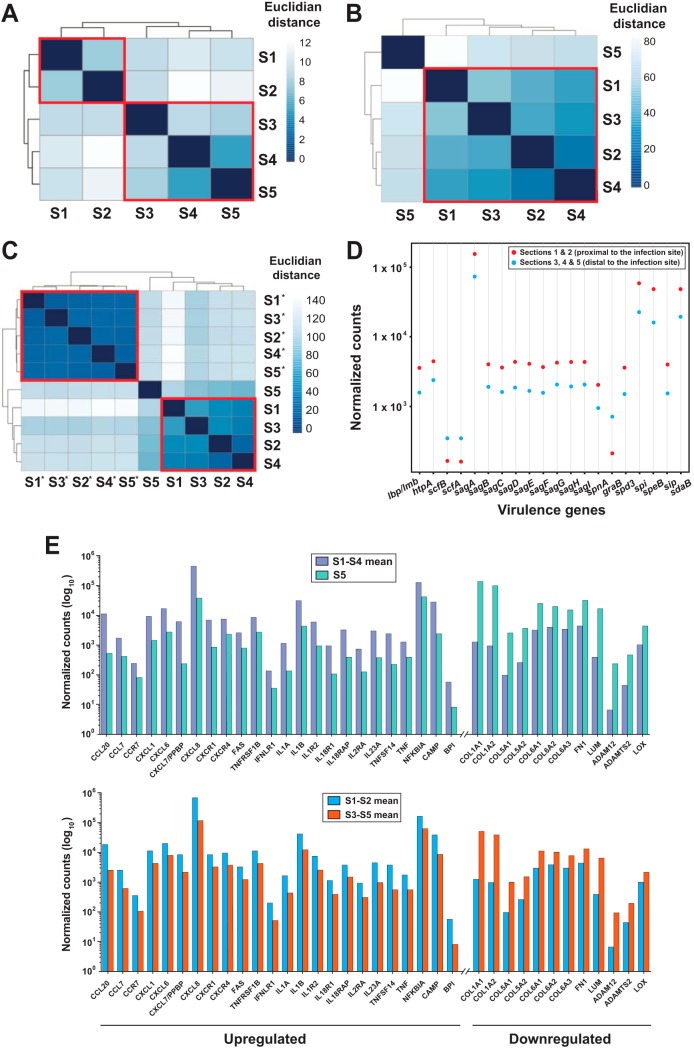
Spatial analysis of GAS and NHP sections. (A) Hierarchical clustering of GAS transcript profiles from individual tissue sections based on Euclidean distance. Data obtained from tissue sections 1 and 2 (closest to the inoculation site) are closely related and cluster into one group, and data from sections 3, 4, and 5 cluster into a second distinct group. (B) NHP transcripts from infected tissue. Data from sections 1, 2, 3, and 4 are closely related and cluster into one group. (C) NHP transcripts from infected and mock-infected tissue. Data from all five sections from the mock-infected tissue, denoted by asterisks, are closely related and cluster into one group. In each case, S1 through S5 correspond to data from analogous sections for the three NHPs pooled and analyzed together as individual units. Each cluster is demarcated by a red perimeter. (D) Differentially expressed GAS virulence genes comparing pooled sections 1 and 2 to sections 3, 4, and 5. Twenty virulence genes were differentially expressed. The fold change value cutoff and adjusted *P* value cutoff were 1.5 and ≤0.05, respectively. (E) Selected significantly upregulated immune response genes are depicted on the left, and selected downregulated extracellular matrix organization and skeletal muscle development genes are shown on the right. (Top panel) Comparison of pooled sections 1 through 4 to section 5. Bottom, comparison of pooled sections 1 and 2 to sections 3 through 5. The fold change value cutoff and adjusted *P* value cutoff were 1.5 and ≤0.05, respectively.

Our experimental design permitted us to test the hypothesis that the GAS and host transcriptomes differ as a function of distance from the inoculation site. To test this hypothesis, we compared the GAS transcriptomes as a function of anatomic relationship to the primary inoculation site. For this spatial (section-to-section) transcriptome analysis, GAS read counts were pooled for each section from the three NHPs. Consistent with the hypothesis, we found that the transcriptomes for sections 1 and 2 differed substantially compared to the transcriptomes for sections 3 through 5 (Fig. 4A). Combining the expression data from sections 1 and 2 (inoculation site) and comparing it to that from pooled sections 3, 4, and 5 (distal site), we found 146 genes to be differentially expressed, with a higher fraction of genes upregulated (∼62%) than downregulated (∼38%) in the inoculation site than in the distal site (Table S1G). Various virulence genes, including genes involved in immune evasion (*htpA*, *lmb*, *sag* operon, *sdaB*, *spd3*, and *spnA*), metal ion homeostasis (*htpA* and *lmb*), tissue destruction (*speB*), and cytotoxicity (*sag* operon), were upregulated, whereas *grab*, *scfA*, and *scfB* were expressed at higher levels at the distal site than at the inoculation site (Fig. 4D). Genes involved in DNA replication, recombination and repair (*polA*, *exoA*, *topA*, *uvrA*, *mutY*, and *ruvA*), cell wall biogenesis (*dacA* and *murA.2*), and metabolic adaptation (malate, citrate, and maltose utilization genes) were also differentially expressed. The data suggest that, depending on location relative to a primary infection nidus, there is likely to be a heterogeneous array of GAS transcriptomes in naturally occurring deep-tissue infection in humans. We thus hypothesized that similar spatial trends might be observed in the NHP transcriptome data.

On the basis of the overall expression profile of the NHP transcriptome, sections 1 to 4 clustered together compared to section 5 (most distal section from the inoculation site) (Fig. 4B). Hence, we combined the expression data from sections 1 to 4, compared the combined data to the data from section 5, and found 1,710 genes to be differentially expressed. Pooled sections 1 to 4 showed significant upregulation of immune response factors and of genes involved in defense and neutrophil activation and degranulation and downregulation of genes involved in extracellular structure organization, indicative of reduced tissue integrity (Fig. 4E, top panel). In a different comparison, NHP sections 1 and 2 (proximal to the inoculation site) were compared to sections 3, 4, and 5, and 899 genes were found to be differentially expressed. Interestingly, those same genes alluded to above were also differentially expressed, although at a lower fold change magnitude (Fig. 4E, bottom panel).

### Genes contributing to GAS fitness in necrotizing myositis: integration of RNA-seq and TraDIS data.

A recent study using the same parental (wild-type) serotype M1 GAS strain (MGAS2221), experimental infection conditions, and transposon-directed insertion site sequencing (TraDIS) method determined a fitness scale for all nonessential genes in NHP necrotizing myositis (46). The availability of these fitness data permitted us to test the hypothesis that a significant relationship exists between the magnitude of *in vivo* fitness (as assessed by TraDIS) and gene transcript remodeling. This relationship has been studied to only a very limited extent in other pathogen-host models, generally with somewhat ambiguous findings (67–69). Our analysis identified a significant relationship between *in vivo* fitness and the *in vitro* RNA-seq data. Comparison of GAS RNA-seq data obtained under *in vivo* conditions to those obtained under *in vitro* conditions (ME and ES phases) found that 43% and 49.6%, respectively, of fitness-related genes identified by TraDIS were significantly differentially expressed (differential fold expression of ≥1.5). These results suggest that a sizeable proportion of genes required for sustaining *in vivo* infection were responding to cues present in host skeletal muscle, resulting in an altered transcript profile.

**FIG 5:**
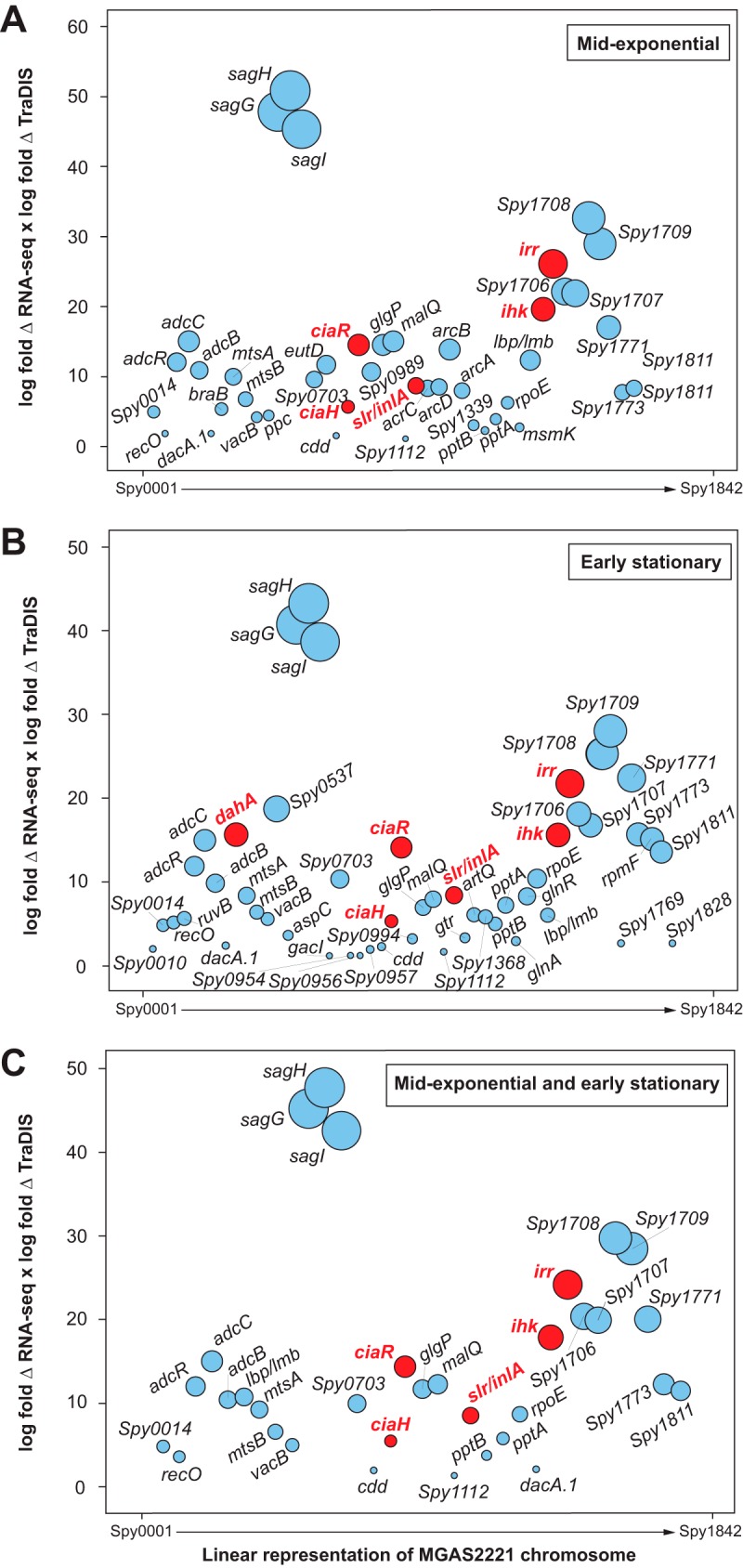
Upregulated GAS genes that contribute to fitness during infection. The *y* axis represents the product of the log-fold change (log-fold Δ) calculated as the product of *in vivo* fold change/*in vitro* fold change, multiplied by the log-fold change calculated in a previous study using the same NHP experimental methods and infecting GAS strain (46). The *x* axis is a linear representation of the GAS chromosome. The size of the circles is proportionate to the product of the 2-fold changes. Red circles represent genes chosen for further study. Gene names shown in red designate genes subsequently studied in more detail by constructing isogenic deletion-mutant strains. The fold change value cutoff and adjusted *P* value cutoff were 1.5 and ≤0.05, respectively. (A) Genes upregulated *in vivo* compared to *in vitro* ME growth phase. (B) Genes upregulated *in vivo* compared to *in vitro* in ES growth phase. (C) Genes upregulated *in vivo* compared to *in vitro* in both ME and ES growth phases.

Of the fitness-conferring genes (i.e., genes that result in significantly decreased fitness when insertionally inactivated) that are also upregulated *in vivo* compared to the ME phase (*n* = 43) or ES phase (*n* = 51), we identified a significant correlation between transcript fold change (log_2_; *in vivo* versus ME or ES phase, dual RNA-seq) and mutant abundance fold change (log_2_; mutant frequency, TraDIS). The significant *P* values computed using a 2-tailed test (Spearman rank correlation) were *r_s_* = 1 and *P = *0 for the TraDIS versus ES comparison and *r_s_* = 0.99 and *P = *0 for the TraDIS versus ME comparison (Fig. 5). Taken together, the data suggest that, on average, GAS genes that are significantly upregulated *in vivo* are likely to be important to pathogen fitness. This idea was tested using isogenic mutant strains and animal infection models as described below.

### Contribution of five new genes to necrotizing myositis virulence: analysis of *Spy0281*, *ihk-irr*, *slr*, *isp*, and *ciaH*.

In principle, the availability of the differential expression data coupled with the TraDIS fitness data affords an enhanced opportunity to exploit this combined information to gain greater understanding of the role of specific genes in pathogen-host interactions, including virulence. There has been little research of this type performed for microbial pathogens. In this context, we elected to study five GAS genes that may participate in necrotizing myositis on the basis of our results here (Table 1; see also Fig. 2, 3, and 5) by constructing isogenic deletion-mutant strains. These five genes were selected based on four criteria: (i) each of the five genes was substantially upregulated *in vivo* in the animal host compared to growth *in vitro*; (ii) we had evidence from a previous genome-wide transposon mutagenesis study (46) suggesting that these genes are important in pathogen-host interactions; (iii) the five genes are all highly conserved among GAS strains; and (iv) no previous study had tested the hypothesis that these genes contribute to necrotizing myositis. Due to cost and other limitations, additional virulence factors were not tested in the present study.

**FIG 6:**
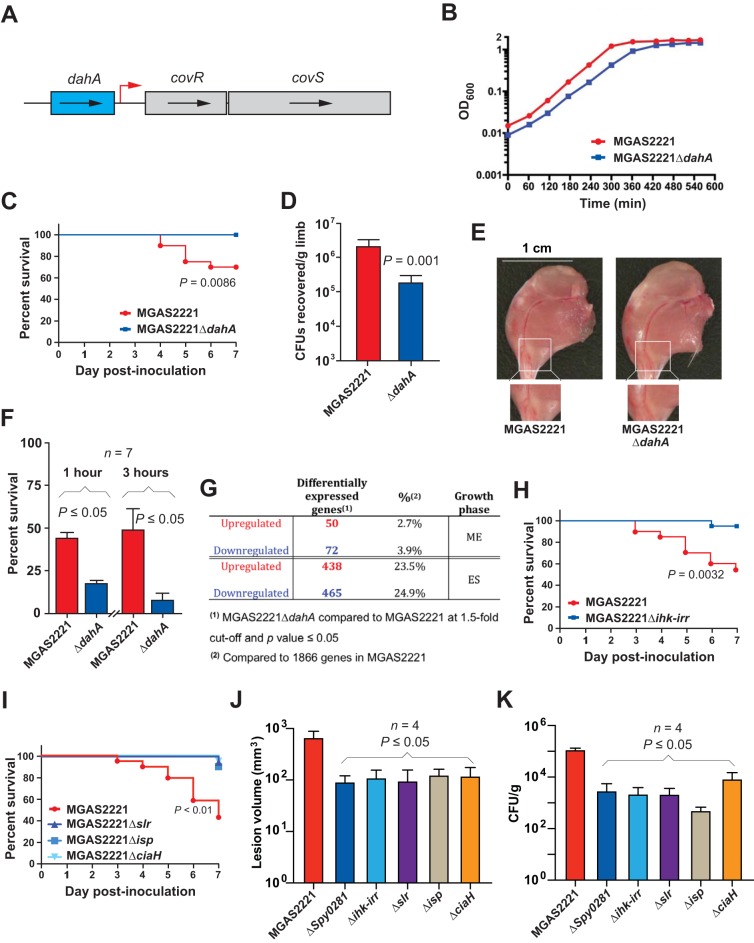
Assessment of virulence of GAS isogenic mutant strains. (A) Schematic showing the *Spy0281* (*dahA*) chromosomal region. *dahA* encodes a putative stress-related protein, and *covRS* encode a two-component system (TCS) involved in virulence. The red arrow marks the transcriptional start site for *covRS* (97). (B) Strains were grown at 37°C in THY medium. (C) Virulence of wild-type MGAS2221 and isogenic mutant MGAS2221 Δ*dahA* in a mouse model of necrotizing myositis (*n *= 20 mice per strain). (D) CFU recovered from the inoculation site of mice. Replicate data (*n *= 35) are expressed as means ± SEM. *P < *0.001, Mann-Whitney *U* test. (E) Representative gross images of mouse hindlimbs infected with the strain MGAS2221 (left) and isogenic MGAS2221 Δ*dahA* strain (right). Lesion areas are boxed in white. Scale bar, 1 cm. (F) MGAS2221 and isogenic mutant strain MGAS2221 Δ*dahA* exposed to purified human PMNs. Percent bacterial survival was assessed at 1 and 3 h as indicated. Means ± SEM of the data from 7 separate experiments are shown. (G) Total number of differentially expressed genes comparing wild-type parental strain MGAS2221 and isogenic mutant strain MGAS2221 Δ*dahA* calculated using a fold cutoff value of ≥1.5 and a *P* value of ≤0.05. (H) Virulence of wild-type MGAS2221 and isogenic mutant MGAS2221 Δ*ihk-irr* in a mouse model of necrotizing myositis (*n *= 20 mice per strain). (I) Virulence of wild-type MGAS2221 and isogenic mutant MGAS2221 Δ*slr*, MGAS2221 Δ*isp*, and MGAS2221 Δ*ciaH* in a mouse model of necrotizing myositis (*n *= 20 mice per strain). (J) Virulence of wild-type and isogenic mutant strains (all derived from parental strain MGAS2221) in an NHP model of necrotizing myositis (*n *= 4 animals per strain). Lesion volume data are expressed as means ± SEM. *P < *0.05, Kruskal-Wallis test. (K) Counts of CFU recovered from the inoculation site of NHPs are expressed as means ± SEM. *P < *0.05, Kruskal-Wallis test. All isogenic mutant strains were derived from parental strain MGAS2221.

First, we studied *Spy0281* (here designated *dahA*, for defense against host protein A), a gene that is differentially upregulated in infected NHPs relative to *in vitro*-grown organisms, at the ME phase of growth. We selected *dahA* for analysis because (i) it is located immediately upstream of genes encoding CovR and CovS, an intensively studied gene regulatory two-component system (TCS) that directly or indirectly influences approximately 15% of the GAS transcriptome (70) (Fig. 6A); (ii) in a previous TraDIS fitness screen, *dahA* was identified as being potentially important during NHP necrotizing myositis (log_2_ fold change = −6.7) (46), and (iii) DahA is homologous to general stress proteins from several bacterial species, but its function in GAS is not known. To test the hypothesis that *dahA* contributes to virulence, we created isogenic deletion-mutant strain MGAS2221 Δ*dahA* and used it in a mouse model of necrotizing myositis. The mutant and wild-type strains had similar growth curves in THY broth (Fig. 6B). However, the mutant strain was significantly less virulent in this infection model (Fig. 6C). Similarly, significantly fewer CFU of the mutant strain than of the wild-type parental strain were cultured from the hindlimbs of infected mice (Fig. 6D), and the resultant lesions were smaller (Fig. 6E). Consistent with the mouse infection data, the isogenic mutant strain was significantly less able to survive exposure to human blood and purified human polymorphonuclear leukocytes (PMNs) than the wild-type parental strain (Fig. 6F). To gain additional information about the role of *dahA*, we conducted RNA-seq analysis of the isogenic strains grown to the ME or ES phase. We discovered that mutant strain MGAS2221 Δ*dahA* had a significantly altered transcriptome compared with the wild-type parental strain (Fig. 6G). Importantly, 17 known virulence genes were significantly downregulated in the isogenic deletion-mutant strain, consistent with the decreased virulence phenotype in the mouse model (Table S1H). Thus, deletion of *dahA* significantly altered the transcriptome, including transcript levels of many virulence genes, strain virulence in mice, and survival when exposed to human PMNs (Fig. 6).

We next examined the role of the *ihk-irr* TCS (71) in necrotizing myositis. This TCS was studied because (i) it was strikingly upregulated *in vivo* (Table S1); (ii) it is important for GAS survival during infection (15, 72–74); (iii) it has been shown previously to be involved in evasion of host innate immune responses, including PMN-mediated killing (75); and (iv) it was identified as contributing to fitness in the TraDIS fitness screen in NHP necrotizing myositis (46). However, the role of *ihk-irr* in necrotizing myositis has not been addressed. To test the hypothesis that *ihk-irr* is involved in necrotizing infection, isogenic mutant strain MGAS2221 Δ*ihk-irr* was used in the mouse model of this disease. Consistent with the hypothesis, the results show that the deletion-mutant strain was significantly less virulent than the wild-type parental strain (Fig. 6H).

We studied a third gene in the context of experimental NHP necrotizing myositis. *slr* (*Spy1109*) encodes an extracellular protein with leucine-rich repeats that is homologous to InlA in the Listeria monocytogenes internalin family of proteins (76). L. monocytogenes InlA anchors the pathogen to the host cell surface by binding E-cadherin and may have a role in bacterial internalization (77, 78). Slr has a domain with histidine triad motifs that is predicted to bind divalent metal cations. Of note, *slr* was shown previously to be significantly upregulated when GAS was grown under conditions of zinc starvation (59). These observations suggest that *slr* is involved in zinc acquisition, a process known to affect GAS virulence (58). We studied the GAS *slr* gene because (i) it was highly upregulated *in vivo* in our dual RNA-seq data (Fig. 3B), (ii) it was identified as contributing to fitness by TraDIS analysis of NHP necrotizing myositis (46), (iii) serologic analysis showed that Slr is expressed *in vivo* in humans with various types of GAS infection (76), and (iv) its role in necrotizing myositis had not been addressed. To test the hypothesis that Slr is involved in necrotizing infection, isogenic mutant strain MGAS2221 Δ*slr* was used in the mouse model of this disease. Consistent with the hypothesis, results show that the deletion-mutant strain was significantly less virulent than the wild-type parental strain (Fig. 6I).

We examined the potential contribution of the *isp* gene to necrotizing myositis. The *isp* gene (encoding an immunogenic secreted protein, or Isp) is located immediately downstream of the *ihk-irr* TCS and encodes a conserved 59-kDa GAS surface protein that induces antibody responses in humans (79). Isp has a CHAP domain located at the carboxy terminus, suggesting that it may be a peptidoglycan hydrolase with a potential role in cell wall metabolism. However, the biological function of Isp is not known, nor is it known if this protein contributes to necrotizing myositis. To test the hypothesis that Isp is involved in necrotizing infection, isogenic mutant strain MGAS2221 Δ*isp* was used in the mouse model. Consistent with the hypothesis, results show that the deletion-mutant strain was significantly less virulent than the wild-type parental strain (Fig. 6I).

The *ciaH* gene encodes the histidine kinase sensor protein of the CiaHR regulator two-component system (TCS). *ciaH* has been reported to be involved in GAS responses to acid and oxidative stresses (80). Interestingly, a previous virulence study found that the *ciaH* deletion mutant was not attenuated in a mouse model of infection, but the route of inoculation was not specified (80). We used the mouse model of necrotizing myositis to test the hypothesis that deletion of *ciaH* significantly decreases virulence. The results demonstrate that the *ciaH* isogenic deletion-mutant strain was significantly attenuated in mice (Fig. 6I).

Inasmuch as GAS is a human-specific pathogen and the *dahA*, *ihk-irr*, *slr*, *isp*, and *ciaH* virulence genes were identified in TraDIS and dual RNA-seq studies performed in NHPs, we sought to confirm the role of each gene in necrotizing myositis using the NHP model. The results showed that each isogenic deletion-mutant strain caused significantly smaller lesions than the MGAS2221 parental wild-type parental strain (Fig. 6J), with significantly lower CFU levels recovered from the infection site (Fig. 6K). These results confirm a role of each of the five GAS genes in experimental necrotizing myositis in primates.

## DISCUSSION

**FIG 7:**
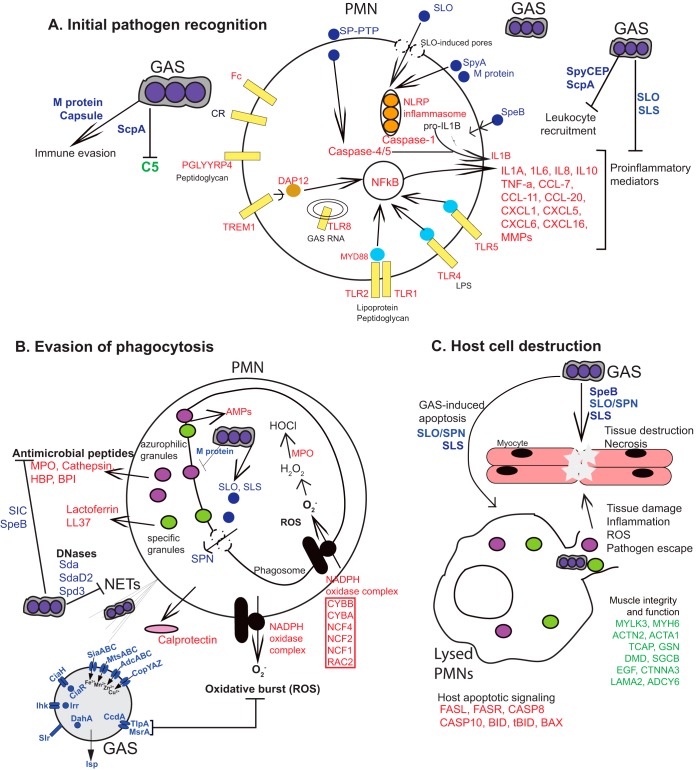
Model of interaction of pathogen- and host-encoded factors to drive GAS-mediated necrotizing myositis pathology. GAS is well adapted to evade host innate immune responses in NHPs and humans. The model is based on new data presented here and includes selected other GAS and host factors crucial for various stages of invasive infection described in the literature, as detailed below. Note that not all pertinent genes and proteins could be included due to space constraints. (A) GAS initiates an acute inflammatory response caused by host recognition of streptococcal components such as lipoteichoic acid and peptidoglycan via extracellular (TLR1, TLR2, TLR4, TLR5, CR, TREM1, and FcR) and intracellular (TLR8) pattern recognition receptors. GAS subverts PMN activation and recruitment by expressing many factors such as hyaluronic acid capsule and M protein and degrading host inflammatory mediators involved in PMN recruitment. (B) GAS evades phagocytosis by producing many antiphagocytic factors (e.g., capsule, M protein). Phagocytosed GAS evade killing by PMNs by secreting cytotoxins (SLO and SPN) and destroying PMNs migrating to the infection site (SLS). GAS-encoded secreted DNases attenuate antimicrobial activity by degrading neutrophil extracellular traps (NETs). Other secreted factors such as streptococcal inhibitor of complement (SIC) and SpeB provide resistance to host antimicrobial peptides (AMPs). In addition, GAS resists host-induced trace metal starvation by upregulating genes involved in acquisition of zinc, iron, copper, and manganese. (C) Host cell destruction and tissue necrosis. GAS factors such as SLO, SLS, and SpeB directly damage cells and induce apoptosis and necrosis via accelerating cell death by triggering activation of proapoptotic host genes. PMN lysis and GAS virulence factors such as SpeB cause skeletal muscle destruction and necrosis as seen by the downregulation of genes involved in skeletal muscle maintenance and integrity. Proteins encoded by upregulated NHP genes are shown in red and downregulated genes in green. Proteins encoded by significantly upregulated GAS genes are shown in blue. AMP, antimicrobial peptide; PRR, pattern recognition receptor; GAS, group A streptococcus; NLRP, NOD-like receptor; CR, complement receptor; FcR, immunoglobulin receptor; TREM-1, triggering receptor expressed on myeloid cells; PGLYRP, peptidoglycan recognition protein; MPO, myeloperoxidase; BPI, bactericidal/permeability-increasing protein; MYLK3, myosin light chain kinase; MYH6, myosin heavy chain; ACTN2, actinin, alpha; ACTA1, actin, alpha; TCAP, titin cap; GSN, gelsolin; DMD, dystrophin; SGCB, sarcoglycan beta; EGF, epidermal growth factor; CTNNA3, catenin alpha; LAMA2, laminin subunit alpha; ADCY6, adenylate cyclase.

Our understanding of the molecular mechanisms used by GAS to initiate and sustain a severe invasive infection, especially during devastating cases of necrotizing myositis, is limited. To address this knowledge deficit, we used a multidimensional investigative strategy employing dual RNA-seq analysis of an extensively documented NHP model of necrotizing myositis (27, 45, 46, 55), coupled with integration of TraDIS fitness data (46) and analysis of isogenic deletion-mutant strains of GAS. Because GAS is a human-specific pathogen, because NHPs are phylogenetically closely related to humans, and because some GAS virulence factors are specific for human or NHP molecules, this animal model best approximates human disease. Our studies identified five new GAS genes encoding virulence factors that significantly contribute to necrotizing myositis. The changes in the NHP transcriptome observed in infected compared to mock-infected animals are consistent with the substantial degree of damaged tissue observed in the animals. In the aggregate, our results significantly enhance understanding of the complex molecular processes transpiring during pathogen-host interactions in necrotizing myositis, knowledge that can be exploited for subsequent basic and translational research activities. A model summarizing the new pathogen and host data presented in this work, together with selected previously reported findings bearing on the molecular mechanisms at work in necrotizing myositis, is presented in Fig. 7.

Dual RNA-seq has been performed on a limited number of pathogens infecting intact hosts (48, 49, 53). In general, previous studies have focused predominantly on descriptive analyses of the resulting data. That is, relatively little new proven gene-specific mechanistic understanding has resulted. As a consequence, we opted to exploit the pathogen transcript data integrated with our previous transposon mutagenesis data to select genes for further analysis using isogenic deletion-mutant strains in subsequent necrotizing myositis pathogenesis studies. Using this integrative and iterative multidimensional strategy, we unambiguously demonstrated that five GAS genes participate in this frequently fatal infection. For example, we showed that *dahA* contributes to adaptation, fitness, and disease in this host environment (Fig. 6). The *dahA* gene is located immediately upstream of *covR* and *covS*, an extensively studied TCS (Fig. 6A) that regulates expression of approximately 15% of the GAS transcriptome when grown *in vitro* (70). Consistent with CovRS being predominantly a negative gene regulator, inactivation of *covRS* increases expression of many genes encoding key virulence factors and enhances disease severity (19, 81). *dahA* is negatively regulated (directly or indirectly) by *covR*, at both the ME and ES phases of growth (82). In addition, *dahA* was identified as contributing to fitness *in vivo* during TraDIS analysis in an NHP model of necrotizing myositis (46). Importantly, 39 genes encoding known virulence factors were differentially expressed *in vitro* in the Δ*dahA* strain compared to the wild-type parental strain (see Table S1H in the supplemental material). Additionally, we found an overlap in upregulated genes between the MGAS2221 Δ*dahA* and MGAS2221 Δ*covR* mutant strains (Table S1I), described in a previous study (82). Seventeen of these genes were downregulated, including some with well-known critical roles in virulence, including *sic*, *emm*, *ropB*, *speB*, *hasABC*, and *mga*. Thus, *dahA* has a complex role in pathogen-host interaction, as many virulence genes are upregulated and many are downregulated. The net effect of the differential *in vivo* gene regulation in a *dahA* mutant is significantly decreased virulence in two animal models of necrotizing myositis. Additional research is under way to further investigate the role of *dahA* in GAS molecular pathogenesis. Similarly, we demonstrated that the *ihk*-*irr* TCS (71) contributes to necrotizing myositis. Our finding adds to previous work showing that this TCS is important for GAS survival during infection, likely as a consequence of altering the expression of multiple genes contributing to innate host defense (15, 72–74). In addition, our data show that the *slr* gene encoding a GAS surface-displayed protein with leucine-rich repeats participates in necrotizing myositis. We previously reported that convalescence-phase serum samples from patients with GAS invasive infections, soft tissue infections, pharyngitis, and rheumatic fever react with Slr (76), indicating that this protein is expressed *in vivo* in infected humans. In the future, it will be important to test the hypothesis that vaccination with one or more of these virulence factors can elicit protective immunity in animal disease models. As stated previously, there is no licensed human S. pyogenes vaccine. As a corollary to these studies, we will test whether one or more of these five virulence factors are potential vaccine candidates via protection studies in mouse or NHP models of infection.

Consistent with the likely situation in humans, we observed some variability in differentially expressed GAS genes between the three NHPs (see Fig. S1D and E in the supplemental material). However, importantly, the animal-to-animal variability was largely due to variation in the magnitude of fold change in individual gene transcripts, rather than to the spectrum of differentially expressed genes. Thus, for all NHPs, the *in vivo* GAS transcriptome is very distinct from that in *in vitro*-grown cells, regardless of growth phase.

With respect to metabolic adaptation, GAS genes encoding enzymes in the Embden-Meyerhof-Parnas (glycolysis) pathway were downregulated *in vivo* whereas genes encoding enzymes involved in transport and use of alternative carbon sources, such as malate and ascorbate (which are abundant in host tissues) and maltose and glycerol, were upregulated (Fig. S3A). Malate utilization is GAS is upregulated *in vitro* under acidic stress conditions and *in vivo* in murine soft tissues (83, 84). This is consistent with data from two recent studies reporting carbohydrate metabolism adaptation and upregulation of production of certain virulence factors during GAS infection (54, 85). Our data are also consistent with GAS undergoing a metabolic switch from homolactic fermentation to mixed-acid fermentation (Fig. S3B).

We observed upregulation of an extracellular oxidative stress defense pathway that uses cytoplasmic thioredoxin as a source of reducing power (Fig. S3C). Broadly similar to a pathway reported for Streptococcus pneumoniae (86), this system is formed by the thiol-disulfide oxidoreductases CcdA and TlpA and the bifunctional methionine sulfoxide reductase MsrA/B, which would protect GAS from the consequences of PMN oxidative burst (87–89). This system may also contribute to GAS extracellular protein folding (90). Importantly, proteins encoded by genes found to be upregulated during infection in our study, including SpeB, SIC (streptococcal inhibitor of complement), and HtpA, are detected in proteomic analyses from human patient samples (91). Taken together, metabolic adjustments made by GAS to the host environment may assist the organism to survive during infection of NHP skeletal muscle.

Our study also addressed a topic germane to many pathogenic microbes. Namely, does a significant relationship exist between the magnitude of *in vivo* fitness and differential regulation of pathogen genes? Previous studies addressing this matter have yielded mixed results (67–69). For example, Turner et al. (68) compared RNA-seq and Tn-seq data sets for P. aeruginosa and reported poor correlation between the two. Comparison of the GAS RNA-seq data generated here with results obtained from a previous genome-wide transposon mutagenesis screen in NHP necrotizing myositis discovered a significant relationship between these two parameters. Our results contrast with (for example) those reported by Powell et al. (92), who studied the relationship between Tn-seq and RNA-seq data for Snodgrassella alvi, a bacterial gut symbiont in honey bees. The investigators found that there was a general lack (6% overlap) of a significant relationship between the RNA-seq and Tn-seq data. One practical implication from our findings is that our multidimensional integrative strategy could be a fruitful way to triage genes for future pathogenesis and translational research. That is, selection of genes for analysis that are highly expressed and display significantly decreased fitness when inactivated and studied in the environment of interest may provide a productive way to discover new pathogenesis clues. Our results reported here on *dahA*, *ihk-irr*, *ciaH*, *isp*, and *slr* provide a proof of principle for this concept and thereby set the stage for additional studies, for example, on genes of unknown function.

Compared to naturally occurring deep-tissue GAS infections in humans, this study was performed under tightly controlled experimental conditions. We used a standard infecting dose of GAS inoculated at the same anatomical site in the three NHPs, and the host tissue samples were collected 24 h later (46, 55). Despite using a standardized infection protocol, we observed some variation in differentially expressed GAS genes between the three NHP hosts, primarily due to variation in the magnitude of fold change in individual gene transcripts, rather than to the spectrum of differentially expressed genes. Furthermore, our spatial (section-to-section) transcriptome analysis yielded variability in gene expression contingent upon anatomic position with respect to the inoculation site. This likely reflects the fact that necrotizing fasciitis/myositis infection outcomes differ from host to host and from tissue to tissue within the same host and might also differ from section to section within the same host tissue, depending on bacterial load (CFU recovered), suggesting that population dynamics and signaling might play a role. Here, we found that some GAS genes encoding or involved in virulence factors, DNA and carbohydrate metabolism, cell wall biogenesis, and dissemination were differentially expressed depending on spatial location with respect to the inoculation site. Importantly, we detected a GAS-associated immunologic response with serum biomarkers in the host (either NHPs or human patients). The levels of several of the tested serum biomarkers were significantly increased in both primate hosts, suggesting that our NHP model of necrotizing myositis mimics the human disease.

Our study used only one GAS strain, a serotype M1 organism that genetically represents a pandemic clone that has emerged relatively recently (27). Given that GAS is characterized by extensive genetic diversity, it is possible that other strains will differ in transcriptome and virulence behavior. We used healthy adult NHPs in this study, with no known immunologic defect. Many patients with GAS necrotizing infections have underlying medical conditions that may influence pathogen-host interactions, including disease character. Thus, it is likely that a more comprehensive understanding of the molecular events involved in this devastating infection would require use of different combinations of GAS strains, host infection sites, and experimental infection protocols.

## MATERIALS AND METHODS

### Bacterial strains and growth media.

For *in vitro* growth, GAS strains were grown in Todd-Hewitt broth (Becton, Dickinson [BD]) supplemented with 0.2% yeast extract (THY medium). This medium was used (i) because it is a standard medium for GAS growth used by many investigators and (ii) because there is no medium that adequately mimics *in vivo* conditions. THY medium was supplemented with spectinomycin (150 μg ml^−1^) as needed. Escherichia coli strains were grown in Luria-Bertani (LB) medium at 37˚C. LB medium was supplemented with spectinomycin (50 μg ml^−1^), as needed. Trypticase soy agar supplemented with 5% sheep red blood cells (Becton, Dickinson and Co.) was also used for growth of GAS strains. Spectinomycin dihydrochloride pentahydrate was purchased from Sigma. Gap electroporation cuvettes (2-mm gap size) were purchased from BTX Harvard apparatus. Growth experiments were performed as described previously (93). Locus tag equivalence between strains MGAS5005 and MGAS2221 is shown in Table S1J in the supplemental material.

### Ethics statement.

All mouse and nonhuman primate studies were performed in accordance with protocols AUP-0318-0016 and AUP-1217-0058 approved by the Institutional Animal Care and Use Committee at the Houston Methodist Research Institute. All studies with human blood and blood components were performed in accordance with a protocol (01-I-N055) approved for human subjects by the Institutional Review Board at the National Institute of Allergy and Infectious Diseases. All study volunteers gave written informed consent.

### Nonhuman primate (NHP) skeletal muscle samples.

We used a well-described NHP model of necrotizing myositis (27, 45, 55). Three cynomolgus macaques (2.8 to 6.5 years of age, 2.9 to 3.5 kg of body weight) were studied. Briefly, the animals were sedated, the injection site was marked, and 1 × 10^8^ CFU/kg serotype M1 strain MGAS2221 was inoculated to uniform depths in the right thigh skeletal muscle. Strain MGAS2221 was used because it has wild-type alleles (i.e., the most common allele) for all major transcriptional regulators, because its genome has been sequenced, because it is genetically representative of contemporary epidemic serotype M1 strains, and because it has been used extensively in animal experiments (27). Animals were observed for 24 h and then subjected to necropsy. The infected thigh muscle was removed *en bloc*, and a 0.5-cm full-thickness transverse section was cut through the injection site with a TissueTek long trimming blade (Bevel). Infected tissue samples weighing ≥150 mg were excised radially in concentric sections from the site of infection and submerged in 2 ml FastPrep tubes (MP Biologicals, Inc.) containing 750 μl of 1× RNA/DNA Shield (ZymoBIOMICS). The samples were collected in duplicate. The sections were numbered 1 through 5, where section 1 corresponds to the inoculation site and 5 to the section most distantly peripheral to the inoculation site (see Fig. S2A in the supplemental material). As a negative control for GAS infection, uninfected skeletal muscle tissue was collected from mock-infected animals given only the same volume of sterile phosphate-buffered saline (PBS). Infected tissue was also collected for quantitative bacterial culture and histopathology examination.

### RNA extraction from NHP tissue.

Two sets of five contiguous tissue sections from the same NHP (see Fig. S3 and S6A) were processed simultaneously. The tissue samples were thawed on ice, and sets of two weighing dishes (24-mm and 85-mm inner base diameter) were wiped with RNaseZap (Invitrogen). The thawed tubes containing the tissue samples were weighed with a Sartorius Entris balance (Fisher Scientific), the tissue was removed with a disposable spatula (VWR) and placed on the small (24-mm-diameter) weighing dish which was then placed inside the larger (85-mm-diameter) dish. The tissue was cut with a disposable scalpel (Exel International), and sections were placed on the larger weighing dish and rubbed against the surface to eliminate excess liquid. The tissue (70 to 90 mg) was washed twice by submerging it in an Eppendorf tube containing 1 ml PBS, gently inverted 10 times to eliminate excess RNA Shield, and placed in a 15-ml Falcon tube (Ambion) containing 3 ml of tissue and cell lysis solution, with 10 μl of proteinase K (50 μg/μl) from a MasterPure complete DNA and RNA isolation kit (Lucigen). Tissues were immediately homogenized (Omni tissue homogenizer; Omni International) on ice at 35,000 rpm, using three bursts of 10 s each. The tubes were incubated at 65°C for 15 min, subjected to vortex mixing every 5 min, and kept on ice for 5 min. The lysate in each tube was transferred to precooled 2-ml tubes containing 0.1-mm-diameter and 0.5-mm-diameter beads (ZR BashingBead lysis tubes; Zymo Research), and bead beating was performed at 1,600 rpm for 60 s and was repeated twice. Between the two bead-beating steps, the tubes were centrifuged at 4°C and immediately placed on ice. The tubes were centrifuged at 4°C at 13,000 rpm for 1 min, and the supernatant was transferred to precooled 1.5-ml Eppendorf tubes. RNA was extracted (MasterPure complete DNA and RNA purification kit; Lucigen) using the manufacturer’s instructions with the exception that the extracted RNA was subjected to three consecutive DNase treatments with a Turbo DNA-free kit (Ambion) at 37°C. RNA quality was assessed with an Agilent 6000 Nano kit (Agilent) and an Agilent 2100 Bioanalyzer. The concentration of total RNA was determined with a Qubit RNA BR assay kit (Thermo Fisher Scientific). Depletion of rRNA was performed with a Ribo-Zero Gold rRNA removal kit, epidemiology (Illumina). The efficacy of the ribosomal depletion procedure was assessed with an RNA 6000 Pico kit (Agilent) and an Agilent 2100 Bioanalyzer.

10.1128/mBio.03363-19.7 FIG S6 Flow charts depicting tissue collection, cDNA sequencing, and transcriptome data analysis. (A) Pipeline representing tissue collection and storage and sample preparation and processing for total RNA extraction, ribodepletion, cDNA library preparation, and cDNA sequencing. The superscript “(a)” indicates that 0.1-mm-diameter and 0.5-mm-diameter beads were used. The superscript “(b)” indicates that the use of the MasterPure protocol with the modification of three DNase treatments, instead of one. The superscript “(c)” indicates the use of a Ribo-Zero Gold rRNA removal kit. (B) Bioinformatic pipeline for demultiplexing, quality assessment, adapter trimming, read mapping to GAS and NHP genomes, data normalization, and differential expression of transcriptome data. Download FIG S6, EPS file, 0.4 MB. Copyright © 2020 Kachroo et al. 2020 Kachroo et al. This content is distributed under the terms of the Creative Commons Attribution 4.0 International license.

### Gene coexpression network analysis.

A weighted gene coexpression network analysis (WGCNA) was performed on the normalized dual RNA-seq section data (*n *= 15 samples) using the WGCNA R software package (66, 94, 95). We largely followed the steps described in the tutorials available on the WGCNA software home page (https://horvath.genetics.ucla.edu/html/CoexpressionNetwork/Rpackages/WGCNA/Tutorials/). To filter out genes expressed at low levels from the analysis, we included only those genes whose normalized counts exceeded 20 in at least 40% of the samples. This resulted in a sample consisting of 1,472 GAS genes and 3,020 NHP genes. The filtered data set was log transformed using log_2_(*x* + 1). To identify outliers, we performed a hierarchical cluster analysis. This revealed one obvious outlier, which was removed from the data. The preprocessed data were split into a pathogen GAS data set and a host NHP data set. We applied WGCNA on the two data sets, using Pearson correlation and the signed hybrid option, to detect GAS- and NHP-specific clusters (or modules) of positively correlated genes. The soft thresholding powers were chosen according to the approximate scale-free topology criterion. The eigengene values of the modules were calculated, and modules were merged if the correlation between their eigengene values exceeded 0.8. COG enrichment analysis of the GAS modules was done in R using Fisher’s exact test and the Benjamini-Hochberg procedure. Pathogen-host interactions were examined by correlating the eigengene values of the GAS and NHP modules.

### Mouse virulence experiments with GAS isogenic mutant strains.

Immunocompetent 5-week-old female CD1 mice (Envigo Laboratories) were used for necrotizing myositis virulence studies as described previously (45). Mice were randomly assigned to treatment groups and inoculated in the right hind limb to a uniform depth with 7.5 × 10^6^ CFU of isogenic mutant strain MGAS2221 Δ*Spy0281* and wild-type parental strain MGAS2221 in 100 ml PBS. Stocks of each strain were prepared at known CFU levels and stored at –80°C. Inocula were prepared by diluting frozen stocks in PBS to the desired number of CFU. For survival experiments (*n *= 20 mice/strain), near-mortality was determined by observation using predefined criteria. For histology evaluation, lesions were excised, visually inspected, and photographed. Histopathology was scored by a pathologist blind to the strain treatment groups as described previously (27, 45). For the quantitative culture experiment (*n *= 35 mice/strain for the wild-type and MGAS2221 Δ*Spy0281* strains), limbs were homogenized (Omni International) and CFU counts were determined by culturing serial dilutions. Identical methods were used to assess the virulence of all additional isogenic deletion-mutant strains. All mouse experiments were approved by the Institutional Animal Care and Use Committee of Houston Methodist Research Institute.

### NHP virulence experiments with GAS isogenic mutant strains.

The NHP model of necrotizing myositis described above and elsewhere (27, 45, 55) was used. Each isogenic deletion-mutant strain was given to four animals. Lesion volume, CFU recovery, and histopathology analyses were performed as previously described (27, 45, 55). All NHP experiments were approved by the Institutional Animal Care and Use Committee of Houston Methodist Research Institute.

### GAS bactericidal activity assay.

Bactericidal activity assays with human PMNs and GAS were performed in accordance with protocol 01-I-N055, approved by the Institutional Review Board for human subjects, National Institute of Allergy and Infectious Diseases. All volunteers gave written informed consent prior to participation in the study. Human neutrophils were isolated from the venous blood of healthy volunteers using a standard method (96). The wild-type MGAS2221 and isogenic MGAS2221 Δ*Spy0281* deletion mutant strains were grown to early mid-exponential phase in THY medium with 5% CO_2_ at 37°C (optical density at 600 nm [OD_600_] = ∼0.5). A total volume of 600 μl containing 10^6^ PMNs, 10^6^ bacteria, 10% autologous serum, and HEPES-buffered RPMI 1640 medium was dispensed into a 1.5-ml Eppendorf tube. Assay mixtures were rotated gently at 37°C for 1 or 3 h, at which time saponin (0.1% final concentration) was added to each assay mixture and the tubes were placed on ice for 15 min. An aliquot of each tube was diluted and plated on THY agar plates for enumeration of CFU. Percent GAS survival was determined by comparison of CFU from assays performed with PMNs to CFU from assays performed without PMNs. Data were analyzed with repeated-measures 1-way analysis of variance (ANOVA) and Tukey’s posttest to correct for multiple comparisons (Prism 6 for Mac OS X, GraphPad Prism; GraphPad Software Inc.).

### Statistical analysis.

Unless otherwise stated, error bars represent standard deviations (SD), and *P* values were calculated using the Fisher exact test, the Mann-Whitney test, the Kruskal-Wallis test, or the log rank test. Differential expression analysis was performed using DESeq2 1.16.1. Genes were considered differentially expressed if the change in the level of expression was greater than 1.5-fold and was associated with an adjusted *P* value (Bonferroni corrected) of <0.05. *P* values for pathways significantly associated with the up- and downregulated host genes were computed by the use of Ingenuity pathway analysis (IPA; version 01-14) with Fisher’s exact test. WGCNA was used with Pearson correlation and the signed hybrid option to detect GAS- and NHP-specific clusters (or modules) of positively correlated genes. The soft thresholding powers were chosen according to the approximate scale-free topology criterion. The eigengene values were calculated for the modules, and modules were merged if the correlation between their eigengene values exceeded 0.8. The enrichment analyses of the modules were done in R using Fisher’s exact test and the Benjamini-Hochberg procedure. For all animal experiments, statistical testing was performed using GraphPad Prism (version 8.1.2). Nonparametric statistical tests were used because the lesion volume and CFU data did not fit a normal distribution using the Anderson-Darling test, the D’Agostino-Pearson test, the Shapiro-Wilk test, or the Kolmogorov-Smirnov test. For mouse survival studies, results were graphed as Kaplan-Meier curves and data were analyzed using the log rank test, with *P* values of <0.05 considered to be significant. For the mouse CFU experiment, data were graphed as means ± standard errors of the means (SEM) and analyzed using the Mann-Whitney test, with *P* values of <0.05 considered to be significant. For the NHP virulence studies, lesion volume and CFU data were graphed as means ± SEM and analyzed using the Kruskal-Wallis test, with *P* values of <0.05 considered to be significant.

### Data availability.

A slightly updated complete genome sequence of *emm1* reference strain MGAS2221 (GenBank accession number CP043530) has been deposited in the NCBI GenBank database under the same accession number. Transcriptome data have been deposited in the Gene Expression Omnibus under accession number GSE144100.
