# Supplementary figures and images for: New Pathogenesis Mechanisms and Translational Leads Identified by Multidimensional Analysis of Necrotizing Myositis in Primates
Source: mBio. 2020 Feb 18;11(1):e03363-19. doi: 10.1128/mBio.03363-19 (PMC7029145; doi:10.1128/mBio.03363-19)

Figure S3A

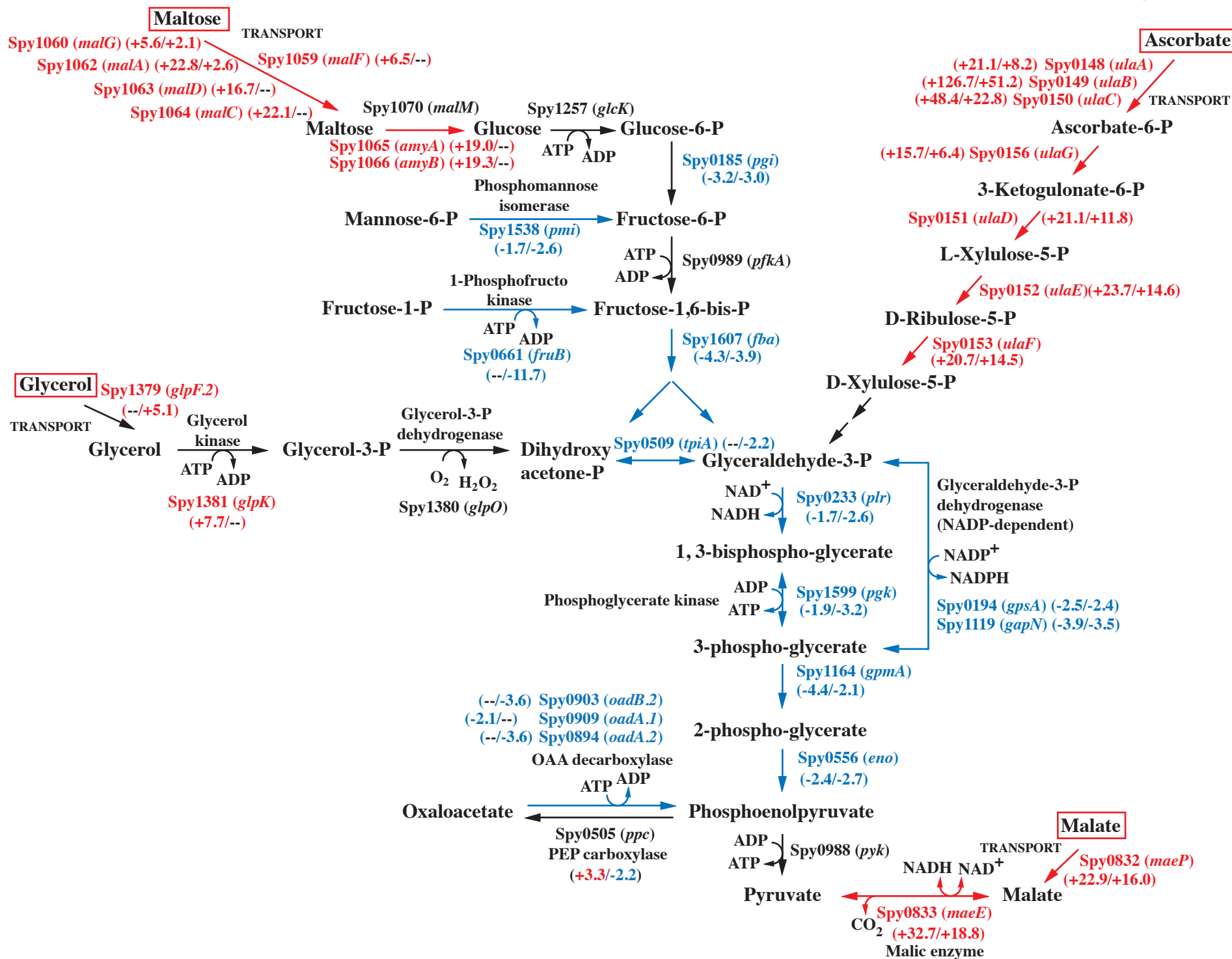

Figure S3B

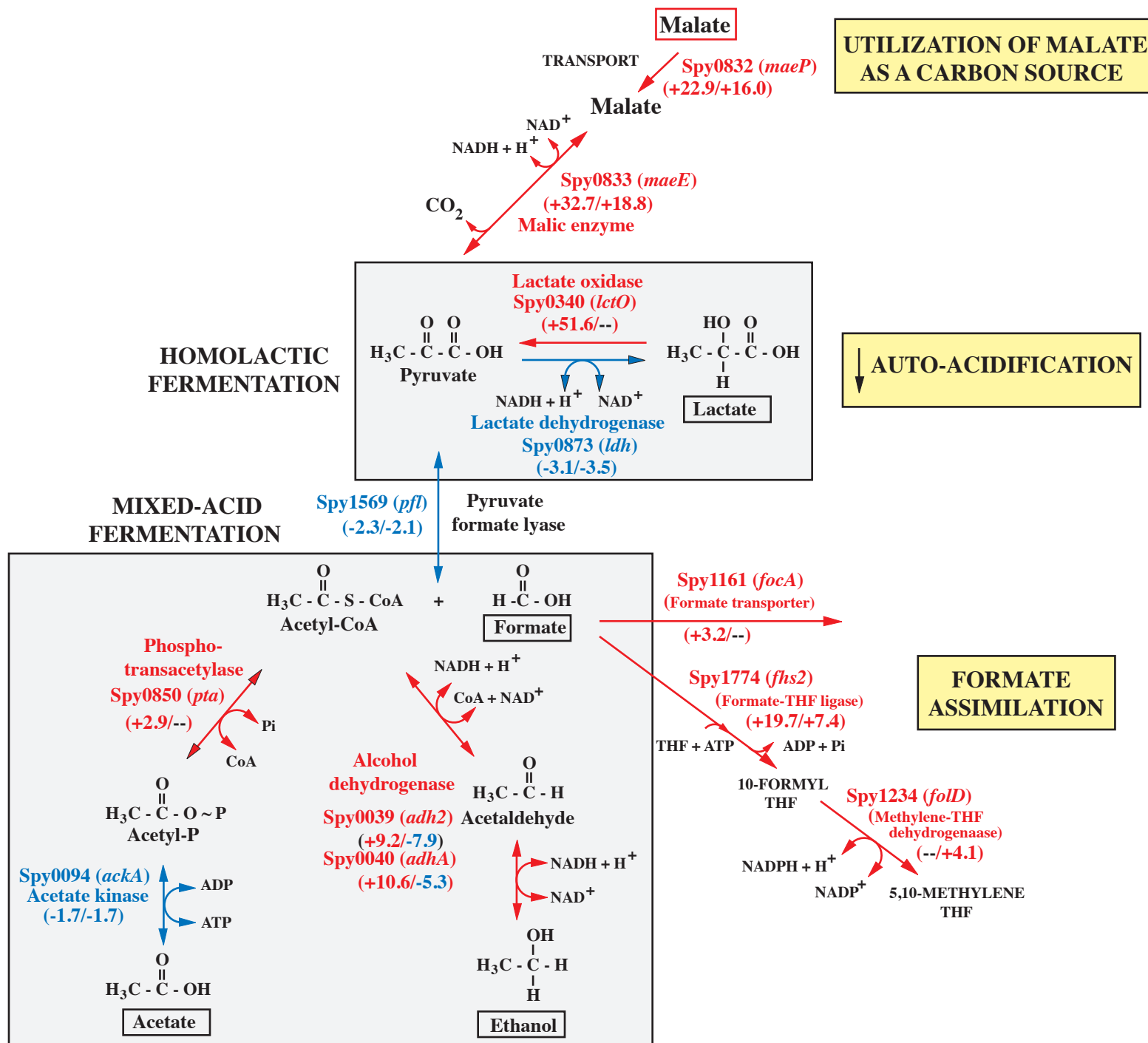

Figure S3C

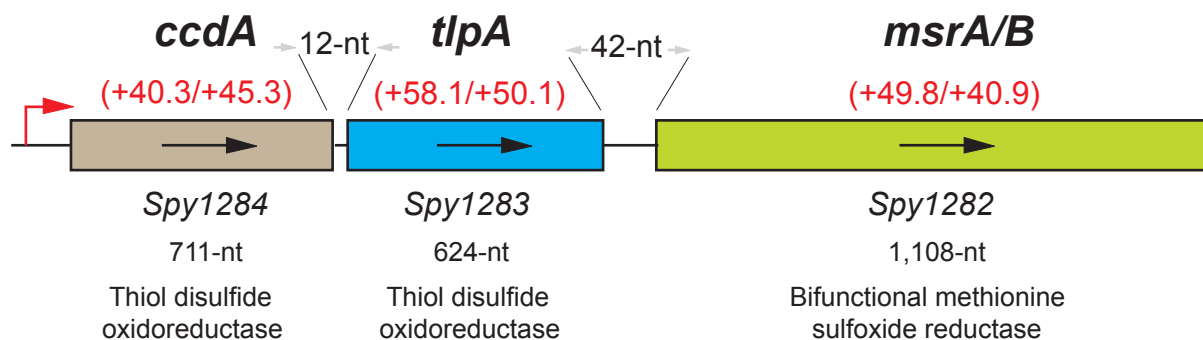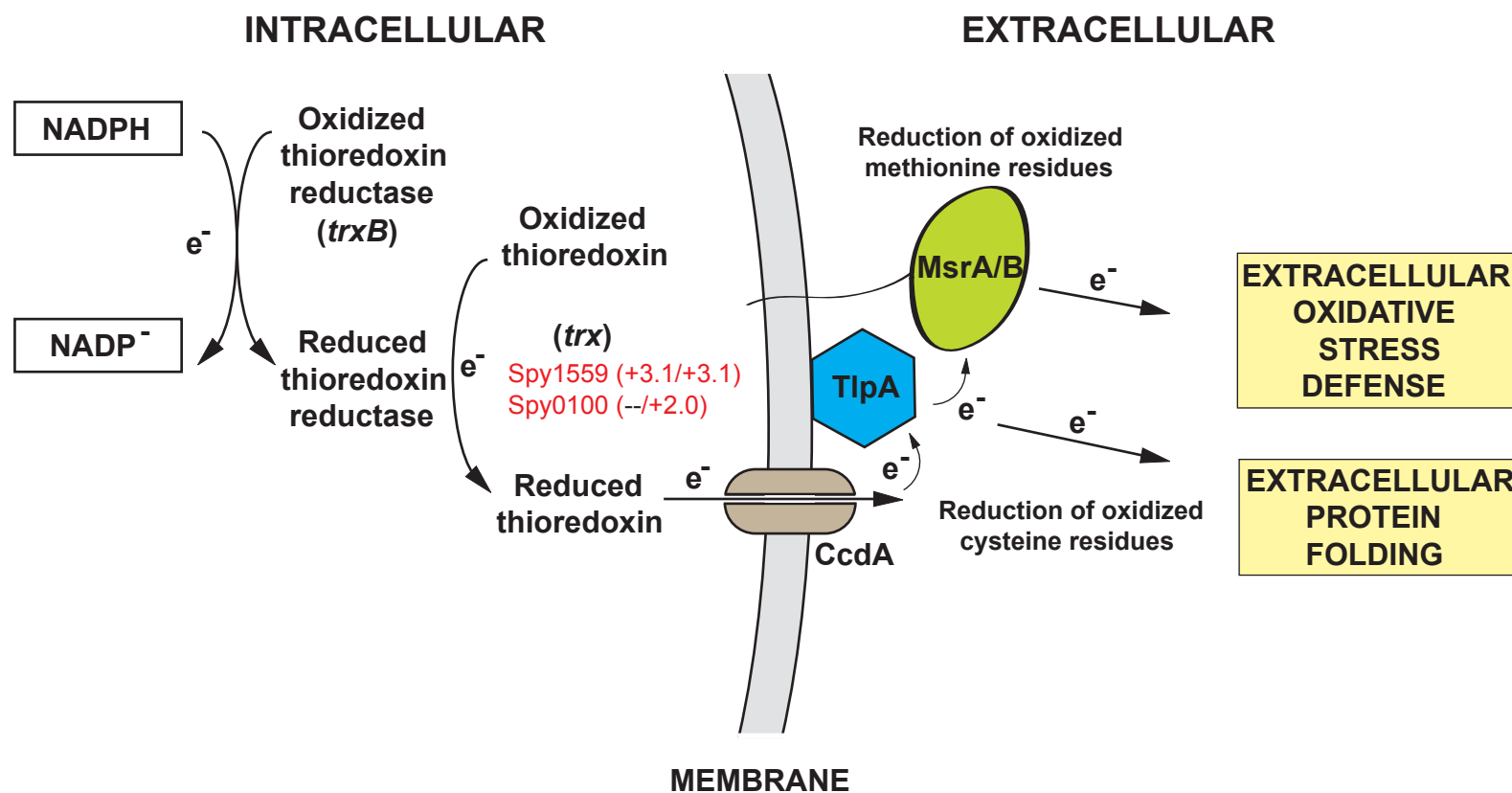

Supplement: FIG S3 [file mBio.03363-19-sf003.pdf]
