# Supplementary material for: New Pathogenesis Mechanisms and Translational Leads Identified by Multidimensional Analysis of Necrotizing Myositis in Primates
Source: mBio. 2020 Feb 18;11(1):e03363-19. doi: 10.1128/mBio.03363-19 (PMC7029145; doi:10.1128/mBio.03363-19)
Supplement: FIG S4 [file mBio.03363-19-sf004.pdf]

**Figure S4A**

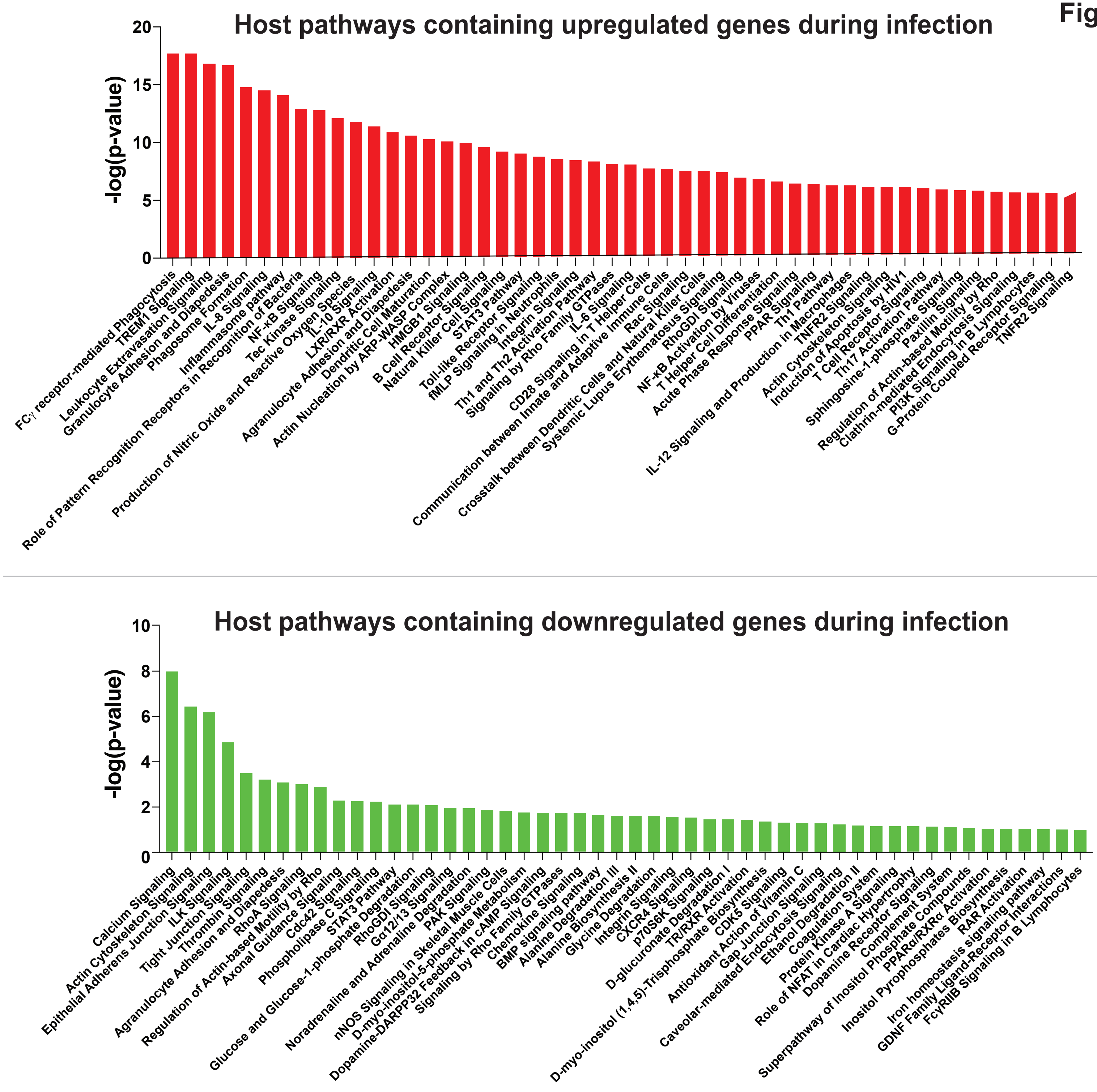
Host pathways containing upregulated genes during infection
-log(p-value)
0
5
10
15
20
FCγ receptor-mediated Phagocytosis
TREM1 Signaling
Leukocyte Extravasation Signaling
Granulocyte Adhesion and Diapedesis
Phagosome Formation
IL-8 Signaling
Inflammasome pathway
Role of Pattern Recognition Receptors in Recognition of Bacteria
NF-κB Signaling
Tec Kinase Signaling
Production of Nitric Oxide and Reactive Oxygen Species
IL-10 Signaling
LXR/RXR Activation
Agranulocyte Adhesion and Diapedesis
Dendritic Cell Maturation
Actin Nucleation by ARP-WASP Complex
HMGB1 Signaling
B Cell Receptor Signaling
Natural Killer Cell Signaling
STAT3 Pathway
Toll-like Receptor Signaling
fMLP Signaling in Neutrophils
Integrin Signaling
Th1 and Th2 Activation Pathway
Signaling by Rho Family GTPases
IL-6 Signaling
CD28 Signaling in T Helper Cells
Communication between Innate and Adaptive Immune Cells
Rac Signaling
Crosstalk between Dendritic Cells and Natural Killer Cells
Systemic Lupus Erythematosus Signaling
RhoGDI Signaling
NF-κB Activation by Viruses
T Helper Cell Differentiation
Acute Phase Response Signaling
PPAR Signaling
IL-12 Signaling and Production in Macrophages
Th1 Pathway
TNFR2 Signaling
Actin Cytoskeleton Signaling
Induction of Apoptosis by HIV1
T Cell Receptor Signaling
Th17 Activation Pathway
Sphingosine-1-phosphate Signaling
Paxillin Signaling
Regulation of Actin-based Motility by Rho
Clathrin-mediated Endocytosis Signaling
PI3K Signaling in B Lymphocytes
G-Protein Coupled Receptor Signaling
TNFR2 Signaling
Host pathways containing downregulated genes during infection
-log(p-value)
0
2
4
6
8
10
Calcium Signaling
Actin Cytoskeleton Signaling
Epithelial Adherens Junction Signaling
ILK Signaling
Tight Junction Signaling
Thrombin Signaling
Agranulocyte Adhesion and Diapedesis
RhoA Signaling
Regulation of Actin-based Motility by Rho
Axonal Guidance Signaling
Cdc42 Signaling
Phospholipase C Signaling
STAT3 Pathway
Glucose and Glucose-1-phosphate Degradation
RhoGDI Signaling
Gα12/13 Signaling
Noradrenaline and Adrenaline Degradation
PAK Signaling
nNOS Signaling in Skeletal Muscle Cells
D-myo-inositol-5-phosphate Metabolism
Dopamine-DARPP32 Feedback in cAMP Signaling
Signaling by Rho Family GTPases
Chemokine Signaling
BMP signaling pathway
Alanine Degradation III
Alanine Biosynthesis II
Glycine Degradation
Integrin Signaling
CXCR4 Signaling
p70S6K Signaling
D-glucuronate Degradation I
D-myo-inositol (1,4,5)-Trisphosphate Biosynthesis
TR/RXR Activation
CDK5 Signaling
Antioxidant Action of Vitamin C
Gap Junction Signaling
Caveolar-mediated Endocytosis Signaling
Ethanol Degradation II
Coagulation System
Protein Kinase A Signaling
Role of NFAT in Cardiac Hypertrophy
Dopamine Receptor Signaling
Superpathway of Inositol Phosphate Compounds
Complement System
Inositol Pyrophosphates Biosynthesis
PPARα/RXRα Activation
Iron homeostasis signaling pathway
RAR Activation
GDNF Family Ligand-Receptor Interactions
FcγRIIB Signaling in B Lymphocytes

**Figure S4B**

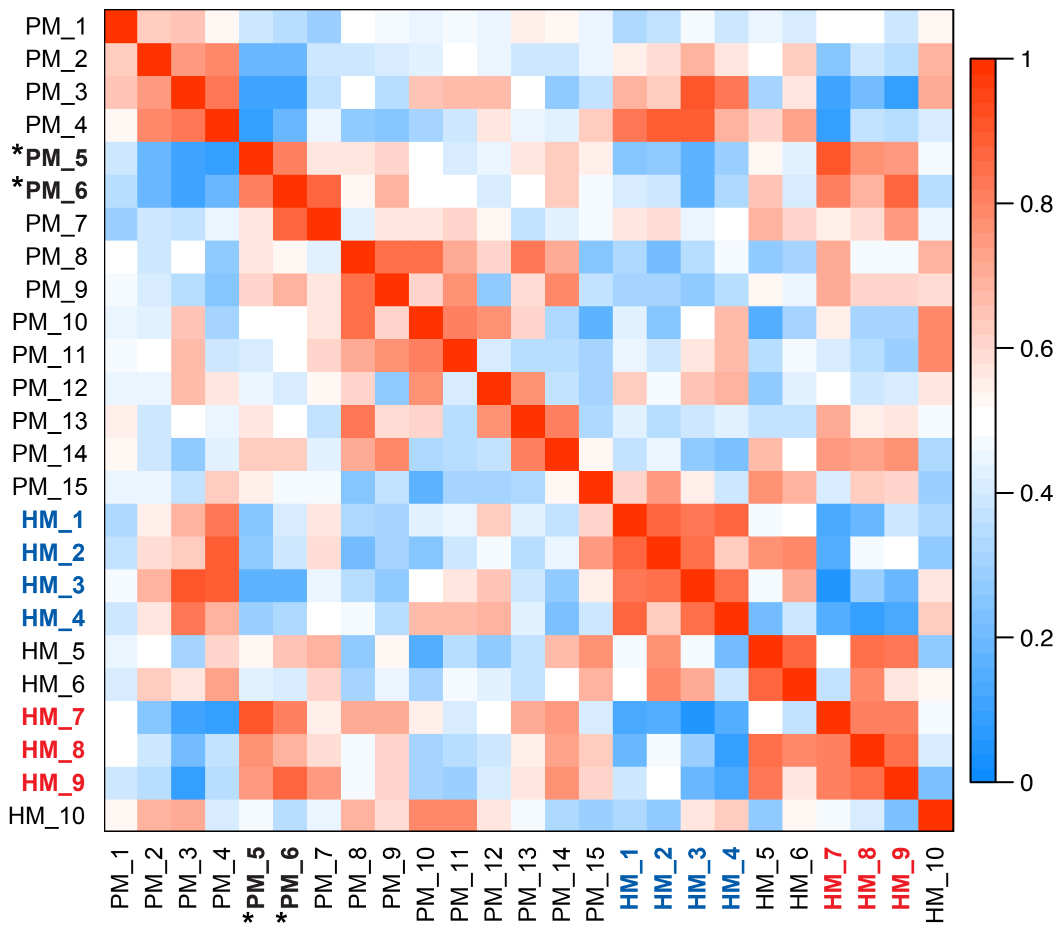


**GAS and host gene modules showing significant correlation**

**PM,** pathogen (GAS) module

**HM,** host module

**PM_5,** involved in carbohydrate transport/metabolism and virulence

**PM_6,** involved in post-translational modifications, protein turnover,chaperones, and virulence

**HM_7,** involved in immune response

**HM_8,** involved in inflammation

**HM_9,** involved in response to stress

**HM_1,** involved in muscle development

**HM_2,** involved in muscle contraction

**HM_3,** involved in muscle energetics

**HM_4,** involved in muscle metabolism
