# Supplementary material for: New Pathogenesis Mechanisms and Translational Leads Identified by Multidimensional Analysis of Necrotizing Myositis in Primates
Source: mBio. 2020 Feb 18;11(1):e03363-19. doi: 10.1128/mBio.03363-19 (PMC7029145; doi:10.1128/mBio.03363-19)
Supplement: TEXT S1 [file mBio.03363-19-s0001.docx]

**SUPPLEMENTAL MATERIALS**

**LIST OF SUPPLEMENTAL FIGURES**

**Fig. S1**. Transcriptome analysis of GAS grown *in vitro* and *in vivo,* and NHP samples.

**Fig. S2**. Sample collection for dual RNA-seq analysis and GAS genes differentially expressed during infection, grouped by functional categories.

**Fig. S3**. Differentially expressed GAS genes implicated in carbon metabolism and extracellular oxidative stress defense in GAS.

**Fig. S4**. Pathway analysis of differentially expressed genes in infected NHP tissue and heatmap depicting relationship between gene co-expression modules.

**Fig. S5**. Biomarker analysis of serum samples from humans and NHPs.

**Fig. S6**. Flow charts depicting tissue collection, cDNA sequencing, and transcriptome data analysis.

**SUPPLEMENTAL FIGURE LEGENDS**

**Fig. S1. Transcriptome analysis of GAS grown *in vitro* and *in vivo,* and NHP samples**. Principal component analyses of transcriptome data. The fold-change and adjusted *P*-value cut-off were 1.5, and ≤ 0.05, respectively. **A**. *In vitro* grown GAS strains in triplicate at mid-exponential (ME) and early stationary (ES) phases of growth, and *in vivo* grown GAS strains collected from three non-human primates (NHP). Five samples (sections 1 through 5) collected from each NHP were pooled together for analysis. The *in vitro* ME replicates clustered together and grouped distinctly away from *in vitro* ES samples, and both ME and ES grouped apart from *in vivo* samples. These three distinct clusters are highlighted within ovals. **B**. *In vivo* grown GAS samples are depicted as separate sections. Five sections corresponding to each NHP are highlighted. Sections are color-coded, whereas NHPs are coded by geometric shapes: NHP-1, circles; NHP-2, triangles; and NHP-3, squares. **C**. *In vivo* grown samples from panel **B** shown at a larger scale. Phages and mobile genetic elements were excluded from the analysis. **D**. Infected and mock-infected samples from three NHPs (NHP-1, blue; NHP-2, red; NHP-3, green). Data from mock-infected samples (hatched squares) and infected animals (non-hatched circles) cluster distantly from each other. **E**. Infected (red) and mock-infected (blue) samples separated by sections. Each section was pooled together from three NHPs. All sections corresponding to the mock-infected tissue cluster distinctly together, away from sections corresponding to infected tissue.

**Fig. S2. Sample collection for dual RNA-seq analysis and GAS genes differentially expressed during infection, grouped by functional categories**. **A**. The samples were processed in concentric sections. Section 1, at the center, corresponds to the inoculation site, and section 5 is the most distal section. The section-to-section borders are idealized and are less discrete than portrayed. Sections from infected tissue are color-coded. Section 1 from the uninfected arm muscle corresponds to the inoculation site, where only PBS was injected. **B**. Differentially expressed (DE) genes *in vivo* versus *in vitro* ME and ES growth phases combined. **C**. DE genes *in vivo* versus *in vitro* ME (non-hatched) and *in vivo* versus *in vitro* ES (hatched). The plots represent the percentage of up- and downregulated genes in each category. Functional categories were obtained from PATRIC (<https://www.patricbrc.org/>) for serotype M1 reference strain MGAS5005. Upregulated genes are represented in red and downregulated genes in blue. The fold-change and adjusted *P*-value cut-off were 1.5, and ≤ 0.05, respectively.

**Fig. S3. Differentially expressed GAS genes implicated in carbon metabolism and extracellular oxidative stress defense in GAS**. Spy numbers correspond to the annotation for serotype M1 reference strain MGAS5005. The fold-change and adjusted *P*-value cut-off were 1.5, and ≤ 0.05, respectively. **A**. Genes encoding glycolytic enzymes are downregulated and genes involved in transport and utilization of ascorbate, malate, maltose, and glycerol are upregulated. Genes differentially expressed *in vivo*, compared to *in vitro*, at ME and/or ES growth phases are colored. Upregulated genes are shown in red and downregulated genes in blue. Fold-changes are represented in parentheses, with the first value corresponding to *in vivo* versus *in vitro* ME and the second value to *in vivo* versus *in vitro* ES. **–** denotes no differential expression. **B**. Shift from homolactic fermentation to mixed-acid fermentation inferred from downregulation of *ldh*, encoding lactate dehydrogenase, and upregulation of *lctO*, encoding lactate oxidase, formate transport and assimilation, and mixed-acid fermentation genes. Reduction in lactate production is likely to result in decreased auto-acidification. **C**. Top. Schematic of the *ccdA*, *tlpA*, and *msrA* operon in serotype M1 GAS. The transcriptional start site (red arrow) was described by Rosinski-Chupin *et al*. (1). The inferred enzymatic functions of the encoded proteins are indicated. The fold upregulation values *in vivo*, compared to both *in vitro* growth conditions, are depicted in parentheses (ME/ES). **–** denotes no differential expression. Bottom. Proposed mechanism for GAS extracellular oxidative stress defense during invasive infections based on data for *Streptococcus pneumoniae* (2). Reducing equivalents (e^-^) from NADPH are channeled through the thioredoxin system in the cytoplasm and transferred outside of the cell across CcdA. In turn, CcdA transfers the reducing equivalents to the thiol-disulfide oxidoreductase TlpA, and through to the bifunctional methionine sulfoxide reductase MsrA. The result is increased protection from oxidation of cysteine residues in secreted proteins and reduction of methionine sulfoxides, both likely occurring as a consequence of PMNs oxidative burst (3-5). This system might also contribute to GAS extracellular protein folding (6).

**Fig. S4. Pathway analysis of differentially expressed genes in infected NHP tissue and heatmap depicting relationship between gene co-expression modules**. **A**. Top 50 pathways associated with differentially expressed genes in the infected NHP tissue compared to mock-infected tissue are shown. y-axis depicts –log(*P*-value). Pathways with *P*-values ≤ 0.05 are considered significant. Host pathways containing genes upregulated (Top) or downregulated (Bottom) during infection. **B**. Heatmap depicts similarity of gene co-expression modules (based on eigengenes adjacency) among and between the 15-pathogen modules (PM) and 10-host modules (HM) identified by WGCNA (7). Eigengenes are module representatives and eigengene adjacency is computed based on their correlation. Heatmap is colored based on adjacency score: red represents high adjacency (positive correlation) and blue represents low adjacency (negative correlation). GAS gene modules 5 and 6 (marked with an *) are positively correlated with host gene modules 7, 8, and 9 (highlighted in red), and negatively correlated with host gene modules 1-4 (highlighted in blue).

**Fig. S5. Biomarker analysis of serum samples from humans and NHPs**. The biomarkers selected for the custom panel were: (1), BAFF/BLyS/TNFSF13B; (2), CTSS/Cathepsin S; (3) CCL7/MCP-3/MARC; (4) CD30/TNFRSF8; (5) CD44; (6) CD163; (7) CXCL2/GRO beta/MIP-2/CINC-3; (8) Fas/TNFRSF6/CD95; (9) IFN-gamma; (10) IL-1 RII; (11) IL-2; (12) IL-8/CXCL8; (13) MMP-1; (14) S100A9; (15) TNF-alpha; (16) TNF RII/TNFRSF1B; and (17) TRAIL R3/TNFRSF10C. Red dots/bars, infected NHPs/patients. Blue dots/bars, uninfected NHP/control human serum. **A**. Serum samples from 17 NHPs collected prior to inoculation with GAS and at the time of necropsy. Wilcoxon matched-pairs signed rank test was used to compute the *P*-values. **B**. Serum samples collected from three infected human patients. and nine healthy, uninfected donors. Statistical significance was computed using Mann-Whitney U test. The Y axis in both panels is represented on a log_10_ scale.

**Fig. S6. Flow charts depicting tissue collection, cDNA sequencing, and transcriptome data analysis**. **A**. Pipeline representing tissue collection and storage, and sample preparation and processing for total RNA extraction, ribodepletion, cDNA library preparation, and cDNA sequencing. ^(a)^, 0.1 and 0.5 mm diameter beads were used; ^(b)^, MasterPure protocol with the modification of three DNase treatments, instead of one; ^(c)^, Ribo-zero gold rRNA removal kit.  **B**. Bioinformatic pipeline for demultiplexing, quality assessment, adapter trimming, read mapping to GAS and NHP genomes, data normalization, and differential expression of transcriptome data.

**LIST OF SUPPLEMENTAL TABLES**

**Table S1**. Differentially expressed GAS genes comparing in vivo and in vitro samples. **A**. Correlation coefficients for transcript data for in vivo and in vitro replicates. **B**. Differentially expressed genes comparing in vivo versus in vitro ME and ES samples combined. **C**. Differentially expressed genes comparing in vivo versus in vitro ME samples. **D**. Differentially expressed genes comparing in vivo versus in vitro ES samples. **E**. Differentially expressed genes encoding transcriptional regulators. **F**. Virulence, metal homeostasis and stress genes differentially expressed in vivo compared to both in vitro ME and ES phases. **G**. Differentially expressed GAS genes comparing pooled sections 1 and 2 to pooled sections 3, 4, and 5. **H**. Differentially expressed virulence genes comparing MGAS2221 and MGAS2221Δ*Spy0281*. **I**. Upregulated genes comparing MGAS2221 to MGAS2221Δ*dahA* and to MGAS2221Δ*covR*. **J**. Locus tag equivalence between MGAS2221 and MGAS5005.

**Table S2**. DE genes and significantly enriched biological processes associated with upregulated and downregulated genes comparing infected versus mock-infected NHPs. **A**. Differentially expressed genes. **B**. Significantly enriched biological processes.

**Table S3**. **A**. Functional categories associated with the host gene modules (HM) identified by WGCNA. **B**. COG enrichment analysis for pathogen modules (PM).

**STUDY DESIGN**

This study was designed to acquire substantial new information about the pathogen and host molecular events occurring during severe necrotizing fasciitis/myositis in primates caused by *S. pyogenes* (GAS). We performed dual RNA-seq to analyze the transcriptomes of GAS and primate skeletal muscle recovered contemporaneously using an extensively documented model of necrotizing fasciitis/myositis (8-11). Our goals were to (i) determine GAS transcriptome signatures during necrotizing fasciitis/myositis infection in primates, compared to growth *in vitro*, (ii) compare global primate gene expression in infected *versus* mock-infected tissue, (iii) analyze interdependence between GAS virulence factor transcripts and infected primate skeletal muscle tissue gene expression, (iv) determine if a correlation exists between the magnitude of GAS virulence gene expression and pathogen fitness, as assessed by genome-wide transposon mutagenesis (11), and (v) analyze the role in virulence of GAS genes found to be important during infection by using isogenic deletion-mutant strains.

The transcriptome of M1 GAS strain MGAS2221 was analyzed at two phases of growth, mid-exponential (ME) and early-stationary (ES), using 3 biological replicates (growth *in vitro*). Three NHPs were injected with GAS, necropsied after 24 h, samples were excised from the site of infection, and analyzed by dual RNA-seq (growth *in vivo*). Mock-infected skeletal muscle tissue was collected as negative control. Virulence studies were performed using two animal models of necrotizing fasciitis/myositis. Mice (*n*=20 mice/strain) were used for near-mortality, and (*n*=35 mice/strain) for CFU determination. In addition, each isogenic deletion-mutant strain was used to infect four NHPs. Lesion volume, CFU recovery and histopathology analysis were used to assess virulence.

**ADDITIONAL METHODS**

**DNA manipulation and analysis**

Standard protocols or the manufacturer’s instructions were used to isolate plasmid DNA for restriction endonuclease digestion, DNA ligase procedures, and PCR and other enzymatic treatments of plasmids and DNA fragments. Enzymes were purchased from New England Biolabs (NEB) Inc. (Beverly, MA). Plasmid DNA was purified with Qiaprep Spin and Qiaquick gel extraction kits (Qiagen). Q5 high-fidelity DNA polymerase was used as the high-fidelity PCR enzyme (NEB). For chromosomal DNA extraction, 800 μl of GAS cells grown to OD ~ 1.0 were centrifuged for 1 min at 16,100 x g, and the pellets were suspended in 800 μl of water. Subsequently, the cells were lysed with a Fastprep 96 MP instrument (Biomedicals, Santa Ana, CA), according to the manufacturer’s instructions. The lysates were centrifuged for 2 min at 16,100 x g, and 600 μl of the supernatant was added to a 1.5-ml Eppendorf containing an equal volume of ice-cold isopropanol. After incubation at -20°C for 2 h, the lysates were centrifuged at 16,100 x g for 30 min, the supernatant was discarded, and the pellets were washed with 70% ice-cold ethanol. After one final high-speed 30 min centrifugation, the supernatant was decanted, and the pellets were dried by incubating the inverted tubes for ~ 30 min. The pellets were dissolved in 50-100 μl of water, and 1 μl of chromosomal DNA was used for subsequent PCR reactions.

DNA sequencing of appropriate clones was performed with a 3730 xl DNA analyzer (Applied Biosystems) and BigDye Terminator v3.1 Cycle Sequencing Kit (LifeTechnologies). Oligonucleotides were purchased from Sigma Genosys. All final clones were sequenced for verification and to rule out the presence of spurious mutations.

**Preparation of cDNA libraries and DNA sequencing**

cDNA libraries were generated from rRNA depleted RNA using a NEBNext Ultra II Directional RNA Library Preparation kit for Illumina (NEB) according to manufacturer’s instructions. The cDNA libraries were prepared with indexed reverse primers from the NEBNext multiplex oligonucleotides from Illumina and purified with SPRIselect beads (Beckman Coulter, Inc.). The quality of cDNA libraries was evaluated with a High Sensitivity DNA kit (Agilent) using an Agilent 2100 Bioanalyzer. The concentration of each sample was measured with Qubit dsDNA Broad Range (BR) and High Sensitivity (HS) kits (Thermo Fisher Scientific). The cDNA libraries were diluted, pooled, and analyzed with an Illumina NextSeq550 instrument.

**Processing and mapping of reads to the genomes of *Macaca fascicularis* and GAS serotype M1 strain MGAS2221**

The bioinformatics pipelines used are shown in fig. S6B. Sequencing read quality was evaluated with FASTQC software (Illumina bcl2fastq, <https://support.illumina.com/downloads/bcl2fastq-conversion-software-v2-20.html>). Adapter contamination and read quality filtering was performed with Trimmomatic (12). Reads were mapped to the genome of *Macaca fascicularis* (5.0, release-95, Ensembl (<http://useast.ensembl.org/Macaca_fascicularis/Info/Annotation>) using STAR (13). Reads mapping to the NHP genome were subsequently mapped to the GAS genome (MGAS2221, Genbank id xxxx). Cross-mapping reads were identified and excluded with SeqFilter (<https://github.com/BioInf-Wuerzburg/SeqFilter/blob/master/README.org>) (Fig. 1). Filtered reads were mapped to the genome of reference GAS strain MGAS2221 using EDGE-pro (14) (<http://ccb.jhu.edu/software/EDGE-pro/>); reads mapping to rRNA and tRNA genes were excluded from subsequent analyses. Differential expression analysis was performed using DESeq2 version 1.16.1. For historical clarity we provide gene designation numbers (*Spy* numbers) for both strain MGAS5005 and MGAS2221 in various tables.

Significant biological processes associated with up- and down-regulated genes were identified with Gene Ontology (GO) Term Finder (<https://go.princeton.edu/cgi-bin/GOTermFinder>). Pathway analysis was performed with Ingenuity Pathway Analysis software, version 01-14 (Qiagen).

**Criteria for exclusion of low-expressed NHP genes**

Transcript reads mapping to the NHP genome were normalized for abundance and gene length. Genes whose mean expression level across the 3 infected and 3 mock-infected samples fell into the lowest 10^th^ quartile (*n* = 2,799 genes) were excluded from further analyses. The vast majority (90%) of these low-expressed genes had zero averaged reads mapping to either the infected or mock-infected samples.

**Construction of isogenic mutant strains**

To create the *Spy0281* deletion mutant, we replaced the entire coding region of *Spy0281* at its original chromosomal locus with the coding region of the *spec* gene from plasmid pJSF84 (15). Thus, the *Spy0281* gene promoter drives the expression of the *spec* gene in the final construct. This strategy allowed for use of a positive selection for the replacement of *Spy0281* through a direct double cross-over recombination event by plating target cells on spectinomycin, as described (8). All original DNA sequences were amplified from wild-type parental strain MGAS2221 chromosomal DNA. Primers JE164 and JE165 were used to amplify a 2,309-bp DNA fragment containing the 534-bp *Spy0281* gene, 875-bp upstream, and 817-bp downstream. The 2,309-bp PCR product was used as template for construction of the deletion mutants using combinatorial PCR (16).

The region located 5’ to *Spy0281* was amplified from the 2,309-bp PCR product generated with the external primers JE164 and JE165 using primers JE164 and JE201; to amplify the region 3’ to *Spy0281* we used primers JE185 and JE165. The 5’ ends of primers JE201 and JE185 contain15 bp that are complementary to the 5’ and 3’ ends of the *spec* gene, respectively. The 753-bp *spec* gene was amplified from pJSF84 using primers JE200 and JE179. These primers contain 15 bp at their 5’-ends complementary to the immediate DNA sequences located upstream and downstream of *Spy0281*. Thus, we generated three contiguous PCR fragments: (i) upstream *Spy0281*, (ii) *spec* gene, and (iii) downstream *Spy0281*. Combinatorial PCR was performed in two sequential steps. First, the upstream *Spy0281* PCR product and the *spec* gene DNA were combined using primers JE164 and JE179. Second, the downstream *Spy0281* PCR fragment was used as a template together with this newly-formed PCR fragment, to generate a new 2,528-bp PCR fragment containing the *spec* gene, instead of *Spy0281*, and the upstream and downstream *Spy0281* sequences, generated using the external JE164 and JE165 primers.

A linear DNA fragment containing contiguous upstream and downstream DNA sequences and the *spec* gene were electroporated into strain MGAS2221, incubated for 2 hr, and plated onto THY agar plates containing spectinomycin. Spectinomycin resistant colonies were selected for further analysis, and PCR amplification of the *Spy0281* DNA region from candidate colonies was performed to verify the chromosomal replacement of *Spy0281* with the *spec* gene using external primer pairs JE164 and JE165. The same DNA regions were also amplified from chromosomal MGAS2221, as control for fragment size difference when electrophoresed on agarose gels.

Isogenic mutant strain MGAS2221Δ*ihk-irr* was generated using allelic exchange. Briefly, primers ihkirr-1 and ihkirr -4 were used to amplify a 4,123-bp fragment using genomic DNA of MGAS2221. Primer sets ihkirr-1 and -2 and ihkirr-3 and -4 were used to amplify two fragments upstream and downstream, respectively, of *ihk-irr*. The two PCR fragments were merged by combinatorial PCR to generate a 2,088-bp fragment containing the *ihk-irr* deletion, which was ligated into the *BamHI* site of suicide vector pBBL740. The recombinant plasmid was transformed into strain MGAS2221 to replace the native *ihk-irr* via allelic exchange, as described previously (17).

Isogenic mutant strains MGAS2221Δ*slr*, MGAS2221Δ*isp* and MGAS2221Δ*ciaH* were generated using allelic exchange and analogous methods described above used to create strain MGAS2221Δ*ihk-irr*. These isogenic mutants contained deletions encompassing 2,019-bp of 2,379 total bp for *slr*, 1,547-bp of 1,602 for *isp*, and 843-bp of 1,314 for *ciaH*. Whole genome sequencing of all mutant strains confirmed the constructs and absence of spurious mutations. The primers used are shown below:

| **Oligonucleotide** | **DNA sequence** |
| --- | --- |
| **JE164** | 5’-GTCGAGGATATGGCTATGGTGGCCCAG-3’ |
| **JE165**  **JE179** | 5’-CCTTGCCTGGAATGTCAATTTTGCCGCGGAG-3’  5’-CTTTAATATTCAGTTTTATAATTTTTTTAATCTGTTATTTAAATAGTTTATAG-3’ |
| **JE185** | 5’-TTAAAAAAATTATAAAACTGAATATTAAAGATGTCTGTAATT TTTATTTG-3’ |
| **JE200** | 5’-ATTAGGAGAAGATGATGAATACATACGAACAAATTAATAAAGTGAAAAAAATAC-3’ |
| **JE201** | 5’-GTTCGTATGTATTCATCATCTTCTCCTAATAAAACTCTCATTTATTTTTACTAC-3’ |
| **Ihkirr-1** | 5’-GTCCGGATCCGAAGAAGAGCTTAGCAAAATACAG-3’ |
| **Ihkirr-2** | 5’-TAGTATGGATCAATCGTCATGATGAGATTGGCGTTTAGTGTATCAAGC-3’ |
| **Ihkirr-3** | 5’-GCTTGATACACTAAACGCCAATCTCATCATGACGATTGATCCATACTA-3’ |
| **Ihkirr-3**  **slr-left-FWD**  **slr-left-REV**  **slr-right-FWD**  **slr-right-REV**  **slr-BBL-FWD**  **slr-BBL-REV**  **ciaH-left-FWD**  **ciaH-left-REV**  **ciaH-right-FWD**  **ciaH-right-REV**  **ciaH-BBL-FWD**  **ciaH-BBL-REV**  **phoZ-bbl-FWD**  **phoZ-bbl-REV**  **isp-ins-FWD**  **isp-ins-REV**  **isp-d1**  **isp-d2** | 5’-GTCCGGATCCTTGGCATTGTTTGGGTTGAAGTCA-3’  5’-GTGAATACATAATTGTCGCAGCTCCAATC-3’  5’-TGACATTTGTCTGGTTAAGTATATCACATCACTTG-3’  5’-ACTTAACCAGACAAATGTCAACCTATCTAAAC-3’  5’-CAATTGTACAGAACAGCAGCTTCTAAAAC-3’  5’-GCTGCTGTTCTGTACAATTGCTAGCGTAC-3’  5’-TGCGACAATTATGTATTCACGAACGAAAATC-3’  5’-GTGAATACATCGGTTCAACAAGTGGTCTC-3’  5’-TATTGACTTGAAACCGCATAATCTGTAAGATAATAATAG-3’  5’-TATGCGGTTTCAAGTCAATAGATCGTTAAGAATG -3’  5’-CAATTGTACATGAAATGGTTTACAAGACG-3’  5’-AACCATTTCATGTACAATTGCTAGCGTAC-3’  5’-TGTTGAACCGATGTATTCACGAACGAAAATC-3’  5’-ATGTATGTAAGAAAACTACAGAAAGTAAAGAATG-3’  5’-AAGCAATGTCCATCAAGCTCTAGTTCAAG-3’  5’-GAGCTTGATGGACATTGCTTGGGCAATC-3’  5’-TGTAGTTTTCTTACATACATTACCTTGTATACCC-3’  5’-CCATCATTGTTTAGGCAAGTATAGAGCAGGCTAGTTTGTTGACCTA-3’  5’-TAGGTCAACAAACTAGCCTGCTCTATACTTGCCTAAACAATGATGG-3’ |

**RNA-seq analysis of strain MGAS2221Δ*Spy0281* isogenic mutant grown *in vitro***

Wild-type MGAS2221 and the MGAS2221Δ*Spy0281* isogenic deletion mutant were grown overnight in 10 ml of THY broth with 5% CO_2_ at 37°C for no longer than 10 hr. Aliquots (0.8 ml) from overnight cultures were used to inoculate 40 ml of pre-warmed THY in 50-ml conical tubes (1:50 dilution) at 37°C. For RNA isolation, strains were collected at two time points: when the optical density (OD_600_) reached 0.5 and 1.65, corresponding to the mid-exponential (ME) and early stationary (ES) phases of growth, respectively. Bacteria from the mid-log (2 ml) and early stationary (1 ml) phases were added to 4 ml, and 2 ml of RNAprotect Bacteria Reagent (Qiagen), respectively, incubated at room temperature for 20 min, and centrifuged at 4,000 rpm for 15 min. The supernatant was discarded, and the bacterial pellet was frozen in liquid nitrogen and stored at -80°C. RNA extraction was performed as described previously (8, 18). The RNA quality of total RNA was evaluated with an Agilent 2100 Bioanalyzer running 2100 Expert software, and RNA Nano chips (Agilent Technologies). For the subsequent steps we used the ScriptSeq Complete kit for bacteria (Illumina), and the rRNA was depleted using its Ribo-Zero magnetic beads. The quality of the rRNA-depleted RNA was evaluated using RNA Pico chips (Agilent Technologies) in an Agilent 2100 Bioanalyzer. The cDNA libraries were prepared using indexed reverse primers from the ScriptSeq Index PCR primers kit (Epicentre), and purified using AMPureXP beads (Agencourt, Beckman Coulter, Inc). The quality of the cDNA libraries was evaluated using DNA high-sensitivity chips (Agilent Technologies) in an Agilent 2100 Bioanalyzer. For each sample, the cDNA library concentration was measured fluorometrically with Qubit™ dsDNA HS Assay Kits (Invitrogen). Adapter contamination was removed from the FASTQ sequence files with Trimmomatic (12) and the RNAseq data were analyzed with the CLC Bio CLC Genomics Workbench 7.0 version (Qiagen). Reads were mapped to the genome of reference strain MGAS5005, excluding rRNA and tRNA genes. Gene expression values from each strain and set of conditions were compared. The Baggerly test (19) and Bonferroni correction were used to test for differential transcript expression. We considered a gene to be differentially expressed when the change in transcript level differed by at least 1.5 fold between strains, and the *P*-value was less than 0.05.

**Determination of the concentration of biomarkers involved in inflammation in serum samples**

Serum samples collected at baseline (prior to inoculation with GAS; n=3) and at the time of necropsy (24 h post-inoculation; n=3) were collected from the three NHPs used in this study, and from 14 additional NHPs with necrotizing fasciitis caused by serotype M1 GAS, using the same experimental conditions. Thus, a total of 17 NHP serum samples were collected at baseline (prior to inoculation with GAS; n=17) and at the time of necropsy (24 h post-inoculation; n=17).

Serum samples were also collected from three human patients (n=3). Samples were collected from one patient with severe necrotizing fasciitis of the lower limb caused by serotype M22 GAS on hospital days 1, 2, 3, 7 and 15 (n=5), and from two additional human patients with GAS necrotizing fasciitis. These studies were approved by the institutional review board at Houston Methodist Hospital and Research Institute (Protocol IRB1010-0199).

Blood from nine healthy donors was obtained after consent to use as a negative control when comparing to serum concentration levels of the human patients. In all, serum samples from the infected NHPs (n=17), human patients (n=3), and healthy controls (n=9) were sent to R&D Systems Biomarker Testing Service (Minneapolis, MN, USA).

Serum samples were diluted (1:2) prior to the assays, and multiplexed serum immunoassays were performed using a custom panel on a Luminex® MAGPIX® reader, following manufacturer’s instructions. The xPONENT® software (Luminex) was used to analyze the data. The biomarkers selected for the custom panel were: (1), BAFF/BLyS/TNFSF13B; (2), CTSS/Cathepsin S; (3) CCL7/MCP-3/MARC; (4) CD30/TNFRSF8; (5) CD44; (6) CD163; (7) CXCL2/GRO beta/MIP-2/CINC-3; (8) Fas/TNFRSF6/CD95; (9) IFN-gamma; (10) IL-1 RII; (11) IL-2; (12) IL-8/CXCL8; (13) MMP-1; (14) S100A9; (15) TNF-alpha; (16) TNF RII/TNFRSF1B; and (17) TRAIL R3/TNFRSF10C. These biomarkers were selected as their corresponding genes were found to be significantly up-regulated in the infected compared to the mock-infected NHP host tissue.

**CONCLUDING COMMENT**

Regardless of discipline, biomedical research strives to discover the molecular mechanisms underlying pathogenesis and disease and exploit this information to enhance human health. In this regard, we think it reasonable to suggest that our findings, together with recent work using proteomic analyses (11, 20), have implications for clinical and translation research aimed at creating new ways to prevent or treat severe invasive necrotizing infections. Thus, there is cause for optimism. However, the magnitude of *in vivo* expression of a large array of well-known and newly-identified virulence genes suggests that our optimism should be tempered and that successful prevention and treatment of established necrotizing infections is likely to continue to be difficult. Successful treatment may also be hindered by the downregulation of several genes involved in GAS cell division, a finding that may provide a partial explanation for the relative lack of curative effect of beta-lactam antibiotics that depend on active cell division for maximum efficacy. The data also may provide insight regarding the periodic success of treating necrotizing fasciitis/myositis with pooled human immunoglobulin preparations, the notion being that neutralizing or opsonic antibodies directed against a wide array of GAS virulence factors are required for successful treatment (21-23). Regardless, the present work, together with genome-wide transposon mutagenesis studies, means that the pathogenesis community has gained extensive new information about the molecular factors contributing to necrotizing myositis.

**SUPPLEMENTAL MATERIALS REFERENCES**

1. Rosinski-Chupin I, Sauvage E, Fouet A, Poyart C, Glaser P. 2019. Conserved and specific features of *Streptococcus pyogenes* and *Streptococcus agalactiae* transcriptional landscapes. BMC Genomics 20:1-15.

2. Saleh M, Bartual SG, Abdullah MR, Jensch I, Asmat TM, Petruschka L, Pribyl T, Gellert M, Lillig CH, Antelmann H, Hermoso JA, Hammerschmidt S. 2013. Molecular architecture of *Streptococcus pneumoniae* surface thioredoxin-fold lipoproteins crucial for extracellular oxidative stress resistance and maintenance of virulence. EMBO Mol Med 5:1852-1870.

3. Sbarra AJ, Karnovsky ML. 1959. The biochemical basis of phagocytosis. I. Metabolic changes during the ingestion of particles by polymorphonuclear leukocytes. J Biol Chem 234:1355-1362.

4. Iyer GYN, Islam, M. F., Quastel, J. H. 1961. Biochemical aspects of phagocytosis. Nature 192:535-541.

5. Rossi F, Zatti M. 1964. Biochemical aspects of phagocytosis in polymorphonuclear leucocytes. NADH and NADPH oxidation by the granules of resting and phagocytizing cells. Experientia 20:21-23.

6. Cho SH, Collet JF. 2013. Many roles of the bacterial envelope reducing pathways. Antioxid Redox Signal 18:1690-1698.

7. Langfelder P, Horvath S. 2008. WGCNA: an R package for weighted correlation network analysis. BMC Bioinformatics 9:559.

8. Eraso JM, Olsen RJ, Beres SB, Kachroo P, Porter AR, Nasser W, Bernard PE, DeLeo FR, Musser JM. 2016. Genomic Landscape of Intrahost Variation in Group A Streptococcus: Repeated and Abundant Mutational Inactivation of the *fabT* Gene Encoding a Regulator of Fatty Acid Synthesis. Infect Immun 84:3268-3281.

9. Nasser W, Beres SB, Olsen RJ, Dean MA, Rice KA, Long SW, Kristinsson KG, Gottfredsson M, Vuopio J, Raisanen K, Caugant DA, Steinbakk M, Low DE, McGeer A, Darenberg J, Henriques-Normark B, Van Beneden CA, Hoffmann S, Musser JM. 2014. Evolutionary pathway to increased virulence and epidemic group A Streptococcus disease derived from 3,615 genome sequences. Proc Natl Acad Sci U S A 111:E1768-1776.

10. Olsen RJ, Sitkiewicz I, Ayeras AA, Gonulal VE, Cantu C, Beres SB, Green NM, Lei B, Humbird T, Greaver J, Chang E, Ragasa WP, Montgomery CA, Cartwright J, Jr., McGeer A, Low DE, Whitney AR, Cagle PT, Blasdel TL, DeLeo FR, Musser JM. 2010. Decreased necrotizing fasciitis capacity caused by a single nucleotide mutation that alters a multiple gene virulence axis. Proc Natl Acad Sci U S A 107:888-893.

11. Zhu L, Olsen RJ, Beres SB, Eraso JM, Saavedra MO, Kubiak SL, Cantu CC, Jenkins L, Charbonneau ARL, Waller AS, Musser JM. 2019. Gene fitness landscape of group A streptococcus during necrotizing myositis. J Clin Invest 129:887-901.

12. Bolger AM, Lohse M, Usadel B. 2014. Trimmomatic: a flexible trimmer for Illumina sequence data. Bioinformatics 30:2114-2120.

13. Dobin A, Davis CA, Schlesinger F, Drenkow J, Zaleski C, Jha S, Batut P, Chaisson M, Gingeras TR. 2013. STAR: ultrafast universal RNA-seq aligner. Bioinformatics 29:15-21.

14. Magoc T, Wood D, Salzberg SL. 2013. EDGE-pro: Estimated Degree of Gene Expression in Prokaryotic Genomes. Evol Bioinform Online 9:127-136.

15. Lukomski S, Hoe NP, Abdi I, Rurangirwa J, Kordari P, Liu M, Dou SJ, Adams GG, Musser JM. 2000. Nonpolar inactivation of the hypervariable streptococcal inhibitor of complement gene (sic) in serotype M1 *Streptococcus pyogenes* significantly decreases mouse mucosal colonization. Infect Immun 68:535-542.

16. Eraso JM, Kaplan S. 2002. Redox flow as an instrument of gene regulation. Methods Enzymol 348:216-229.

17. Ramirez-Pena E, Trevino J, Liu Z, Perez N, Sumby P. 2010. The group A Streptococcus small regulatory RNA FasX enhances streptokinase activity by increasing the stability of the ska mRNA transcript. Mol Microbiol 78:1332-1347.

18. Beres SB, Kachroo P, Nasser W, Olsen RJ, Zhu L, Flores AR, de la Riva I, Paez-Mayorga J, Jimenez FE, Cantu C, Vuopio J, Jalava J, Kristinsson KG, Gottfredsson M, Corander J, Fittipaldi N, Di Luca MC, Petrelli D, Vitali LA, Raiford A, Jenkins L, Musser JM. 2016. Transcriptome Remodeling Contributes to Epidemic Disease Caused by the Human Pathogen *Streptococcus pyogenes*. MBio 7.

19. Baggerly KA, Deng L, Morris JS, Aldaz CM. 2003. Differential expression in SAGE: accounting for normal between-library variation. Bioinformatics 19:1477-1483.

20. Edwards RJ, Pyzio M, Gierula M, Turner CE, Abdul-Salam VB, Sriskandan S. 2018. Proteomic analysis at the sites of clinical infection with invasive *Streptococcus pyogenes*. Sci Rep 8:1-9.

21. Cocanour CS, Chang P, Huston JM, Adams CA, Jr., Diaz JJ, Wessel CB, Falcione BA, Bauza GM, Forsythe RA, Rosengart MR. 2017. Management and Novel Adjuncts of Necrotizing Soft Tissue Infections. Surg Infect (Larchmt) 18:250-272.

22. Parks T, Wilson C, Curtis N, Norrby-Teglund A, Sriskandan S. 2018. Polyspecific Intravenous Immunoglobulin in Clindamycin-treated Patients With Streptococcal Toxic Shock Syndrome: A Systematic Review and Meta-analysis. Clin Infect Dis 67:1434-1436.

23. Reglinski M, Sriskandan S. 2019. Treatment potential of pathogen-reactive antibodies sequentially purified from pooled human immunoglobulin. BMC Res Notes 12:1-6.
