# Supplementary material for: New Pathogenesis Mechanisms and Translational Leads Identified by Multidimensional Analysis of Necrotizing Myositis in Primates
Source: mBio. 2020 Feb 18;11(1):e03363-19. doi: 10.1128/mBio.03363-19 (PMC7029145; doi:10.1128/mBio.03363-19)
Supplement: TABLE S3 [file mBio.03363-19-st003.pdf]

Table S3A. Functional categories associated with the host gene modules (HM) identified by WGCNA

Host Gene Module (HM)	Functional category	Enrichment FDR[(1)]
1	Muscle cell differentiation	4.90E-14
	Myofibril assembly	6.20E-14
	Muscle structure development	6.20E-14
	Muscle system process	1.10E-13
	Muscle cell development	4.40E-13
2	Striated muscle contraction	1.30E-09
	Muscle filament sliding	1.80E-09
	Actin-myosin filament sliding	1.80E-09
	Muscle contraction	2.50E-09
	Actin-mediated cell contraction	2.20E-08
3	Oxidative phosphorylation	1.40E-70
	Cellular respiration	4.30E-62
	Generation of precursor metabolites and energy	1.30E-57
	Mitochondrial ATP synthesis coupled electron transport	4.30E-57
	ATP synthesis coupled electron transport	6.10E-57
4	Carbohydrate metabolic process	1.30E-07
	Cellular catabolic process	2.10E-04
	Catabolic process	4.10E-04
	Generation of precursor metabolites and energy	6.40E-04
	Regulation of cellular catabolic process	1.10E-03
5	Extracellular matrix organization	2.40E-02
	Extracellular structure organization	2.40E-02
6	Translation	2.60E-15
	Peptide metabolic process	2.60E-15
	Peptide biosynthetic process	3.80E-15
	Amide biosynthetic process	6.90E-14
	Cellular amide metabolic process	5.20E-13
7	Cell activation	5.20E-42
	Leukocyte activation involved in immune response	8.40E-40
	Cell activation involved in immune response	8.40E-40
	Myeloid leukocyte activation	1.20E-39
	Leukocyte activation	1.30E-39
8	Cell activation involved in immune response	3.50E-12
	Leukocyte activation involved in immune response	3.50E-12
	Neutrophil mediated immunity	4.30E-12
	Granulocyte activation	6.10E-12
	Neutrophil activation involved in immune response	6.80E-12
9	Response to type I interferon	5.80E-13
	Type I interferon signaling pathway	5.80E-13
	Cellular response to type I interferon	5.80E-13
	Cytokine-mediated signaling pathway	4.60E-11
	Innate immune response	8.70E-10
10	No significant enrichment	NA

(1) **FDR**, False Discovery rate.

Table S3B. COG enrichment analysis for pathogen modules (PM)

	C	G	I	J	L	O	S	V
Module 1	1.000	1.000	1.000	1.000	1.000	1.000	0.839	1.000
Module 2	1.000	1.000	1.000	1.000	1.000	0.381	0.409	1.000
Module 3	0.999	0.999	0.999	**0.002**	0.999	0.999	0.999	0.782
Module 4	0.978	0.978	0.541	0.978	0.453	0.541	0.864	0.978
**Module 5**	0.837	**0.000**	1.000	1.000	1.000	1.000	1.000	**0.009**
**Module 6**	0.469	0.588	1.000	1.000	1.000	**0.006**	1.000	**0.001**
Module 7	**0.005**	0.994	**0.001**	**0.042**	0.994	0.994	0.994	0.994
Module 8	1.000	0.219	**0.059**	1.000	**0.059**	1.000	1.000	**0.059**
Module 9	1.000	1.000	1.000	1.000	0.837	1.000	0.528	1.000
Module 10	1.000	1.000	1.000	1.000	1.000	1.000	**0.079**	1.000
Module 11	1.000	1.000	1.000	**0.063**	0.808	1.000	1.000	1.000
Module 12	0.996	0.996	0.996	0.996	0.996	0.996	0.614	1.000
Module 13	0.993	0.906	0.906	1.000	0.906	0.906	0.906	0.906
Module 14	0.842	0.153	1.000	1.000	1.000	1.000	1.000	1.000
Module 15	**0.034**	1.000	1.000	1.000	1.000	1.000	1.000	1.000

C, Energy production and conversion

G, Carbohydrate transport and metabolism

I, Lipid transport and metabolism

J, Translation, ribosomal structure and biogenesis

L, Replication, recombination and repair

O, Post-translational modification, protein turnover, and chaperones

S, Function unknown

V, Defense mechanisms (Virulence)

p-value thresholds: 0.01 (red), 0.05 (orange), and 0.1 (yellow).
